# DNA Molecular Computing with Weighted Signal Amplification for Cancer miRNA Biomarker Diagnostics

**DOI:** 10.1002/advs.202416490

**Published:** 2025-04-11

**Authors:** Hongyang Zhao, Yumin Yan, Linghao Zhang, Xin Li, Lan Jia, Liang Ma, Xin Su

**Affiliations:** ^1^ State Key Laboratory of Organic‐Inorganic Composites Beijing Advanced Innovation Center for Soft Matter Science and Engineering Beijing Key Laboratory of Bioprocess College of Life Science and Technology Beijing University of Chemical Technology Beijing 100029 China; ^2^ Department of thoracic surgery Tianjin Chest Hospital Tianjin 300222 China; ^3^ Department of Kidney Disease and Blood Purification The Second Hospital of Tianjin Medical University Tianjin 300211 China; ^4^ Clinical Laboratory China‐Japan Friendship Hospital Beijing 100029 China

**Keywords:** DNA computing, machine learning, miRNA, NSCLC diagnostics, signal amplification

## Abstract

The expression levels of microRNAs (miRNAs) are strongly linked to cancer progression, making them promising biomarkers for cancer detection. Enzyme‐free signal amplification DNA circuits have facilitated the detection of low‐abundance miRNAs. However, these methods may neglect the diagnostic value (or weight) of different miRNAs. Here, a molecular computing approach with weighted signal amplification is presented. Polymerase‐mediated strand displacement is employed to assign weights to target miRNAs, reflecting the miRNAs’ diagnostic values, followed by amplification of the weighted signals using localized DNA catalytic hairpin assembly. This method is applied to diagnose miRNAs for non‐small cell lung cancer (NSCLC). Machine learning is used to identify NSCLC‐specific miRNAs and assign corresponding weights for optimum classification of healthy and lung cancer individuals. With the molecular computing of the miRNAs, the diagnostic output is simplified as a single channel of fluorescence intensity. Cancer tissues (*n* = 18) and adjacent cancer tissues (*n* = 10) are successfully classified within 2.5 h (sample‐to‐result) with an accuracy of 92.86%. The weighted amplification strategy has the potential to extend to the digital detection of multidimensional biomarkers, advancing personalized disease diagnostics in point‐of‐care settings.

## Graphical abstract

A molecular computing approach is presented with weighted signal amplification. Polymerase‐mediated strand displacement is employed to assign weights to target miRNAs, reflecting the miRNAs’ diagnostic values, followed by amplification of the weighted signals using localized DNA catalytic hairpin assembly. Cancer tissues (*n* = 18) and adjacent cancer tissues (*n* = 10) are successfully classified within 2.5 h (sample‐to‐result) with an accuracy of 92.86%.

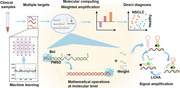

## 1 Introduction

Lung cancer, potentially caused by smoking, air pollution, and heredity, is the leading cause of morbidity and mortality from malignant tumors in the world, accounting for ≈11.4% of the overall cancer incidence.^[^ ^1^ ^]^ For patients with stage I and II lung cancer, the 5‐year survival rate can exceed 80%.^[^ ^2^ ^]^ Detection methods that can rapidly and accurately analyze relevant biomarkers in biological samples from lung cancer patients will greatly facilitate the early diagnosis, monitoring, treatment, and prognosis of lung cancer.^[^ ^3^, ^4^, ^5^ ^]^ In comparison to two biomarkers, proteins, and genomic DNA, miRNAs are involved in almost every stage of cancer development.^[^ ^6^, ^7^ ^]^ Consistent and reproducible differences in miRNA expression levels have been reported between cancer patients and healthy controls. Research indicates miRNA aberrant expression in 8 major cancer types.^[^ ^8^ ^]^ In NSCLC, 161 miRNAs show abnormal expression. Different miRNAs show different changing trends. For example, miR‐21 has the highest expression levels in stage I and stage II.^[^ ^9^ ^]^ Moreover, there are reports indicating that the dysregulation of the miRNA biosynthesis pathway is associated with poor prognosis in NSCLC.^[^ ^10^, ^11^ ^]^ Therefore, miRNA is an important cancer biomarker for rapid and stable diagnosis of lung cancer.^[^ ^12^ ^]^ Over 1000 distinct miRNAs have been identified in the human body.^[^ ^13^ ^]^ Their concentrations can vary widely depending on the biological matrix (e.g., blood, plasma, serum, urine, saliva), tissue type, physiological state, and disease condition. This variability reflects the highly dynamic regulation and multifaceted roles of miRNAs in gene expression, cell signaling, and disease progression.^[^ ^14^ ^]^ For instance, certain miRNAs such as miR‐34a can reach concentrations of up to 1 nm in tissue, while their levels in circulation may fall below the femtomolar (fm) range.^[^ ^15^ ^]^ Notably, miR‐21, a well‐established oncogenic miRNA, is found at significantly elevated levels in various cancer types—often 10‐ to 100‐fold higher than in healthy individuals.^[^ ^16^ ^]^ In NSCLC, for example, miR‐21 levels are approximately ten times higher in stage III–IV tumors compared to stage I–II.^[^ ^17^ ^]^ Currently, miRNA detection methods mainly include reverse transcription quantitative PCR, next‐generation sequencing, and microarray, which can provide quantitative analysis of miRNA expression profiles for cancer diagnosis.^[^ ^18^ ^]^ Although these techniques are capable of quantitative or semi‐quantitative analysis of miRNA, they usually require expensive equipment, cumbersome protocols, and skilled technicians, and are not conducive to clinical dissemination. Moreover, the test results output by the above methods are difficult to use directly for diagnosis.^[^ ^19^ ^,^ ^20^ ^]^

**Figure 1:**
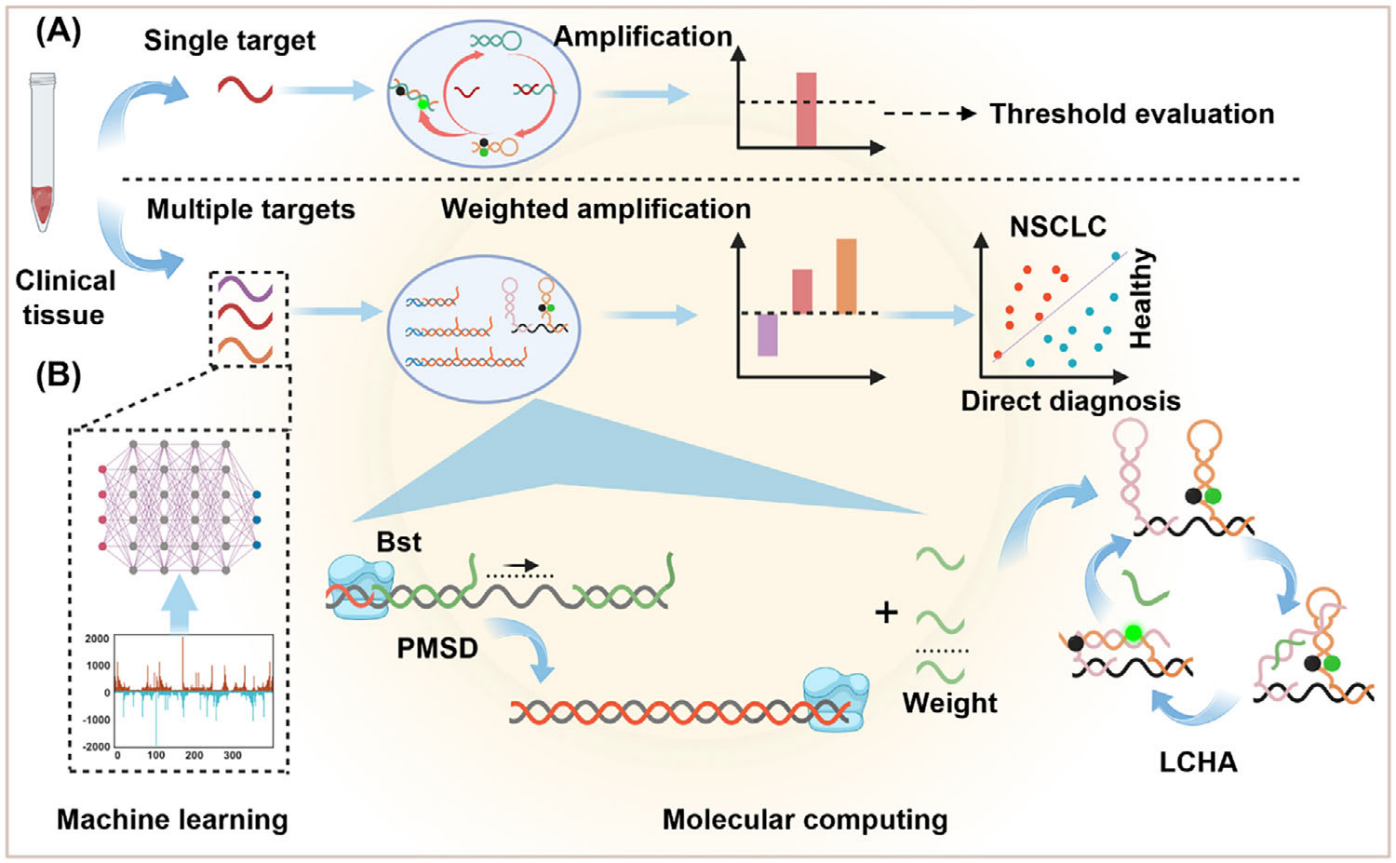
Weighted amplification molecular computing strategy for cancer diagnosis. A) Traditional isothermal signal amplification techniques achieve signal enhancement by circulating targets. However, these techniques only focus on amplifying a single miRNA target and the output results are difficult to be used directly for diagnosis. B) We integrated PMSD and LCHA reactions to the biological information provided by the differential expression of multiple miRNAs, finally realizing direct diagnosis. According to the machine learning screened miRNAs and their weight information, PMSD assigns weights for multiple miRNA biomarkers by releasing weight DNA strands, and LCHA amplifies these weight DNA strands to achieve a detectable fluorescence signal. Finally, this strategy was used for NSCLC tissue sample diagnosis.

With the development of dynamic DNA nanotechnology,^[^ ^21^, ^22^, ^23^, ^24^ ^]^ since the concept of Hybridization Chain Reaction (HCR) was first introduced by Dirks and Pierce in 2004,^[^ ^25^ ^]^ subsequent isothermal technologies such as Entropy Driven Catalysis (EDC), Catalytic Hairpin Assembly (CHA), which achieve signal amplification by hybridization chain reaction or cycling input for sensitive miRNA detection.^[^ ^26^, ^27^, ^28^ ^]^ The introduction of immobilization techniques, such as localized CHA (LCHA), where two reaction hairpins are immobilized on a DNA tile, confines the spatial distance to nanometers, which corresponds to an increase in the concentration of the reaction hairpins, and thus increases the reaction rate of the probes.^[^ ^29^, ^30^ ^]^ However, these signal amplification techniques focus only on the amplification of single targets ignoring the biological information provided by the differential expression of multiple miRNAs in clinical samples. The output results are difficult to use for direct diagnosis (**Figure** **1**A).^[^ ^31^, ^32^ ^]^ Therefore, it is motivated to develop a method for detecting this biological information.

DNA molecular computing technology combines a series of DNA molecular reactions with logical analysis, allowing for different logical connections (AND, OR, NOT) and algorithms (addition, subtraction, weighting) at the molecular level.^[^ ^33^, ^34^, ^35^, ^36^ ^]^ It has been applied to a variety of intelligent diagnostics, such as distinguishing bacterial infections from viral infections and classifying cancer as healthy.^[^ ^37^ ^]^ However, the current DNA computing system utilizes the chemistry of a seesaw gate which requires a large number of DNA strands to achieve addition, subtraction, and weighting operations.^[^ ^37^, ^38^, ^39^ ^]^ This makes it difficult to integrate such isothermal signal amplification gates, limiting their capability to directly sense targets in clinical samples.

In this study, we proposed a weighted amplification molecular computing strategy for NSCLC miRNA diagnostics (Figure 1B). Screening miRNAs specific to NSCLC from specialized databases and assigning them appropriate weights can provide a solid foundation for enhancing diagnostic accuracy. A support vector machine (SVM) algorithm was used to screen signature miRNAs from publicly available miRNA‐seq profiles of healthy and NSCLC individuals from The Cancer Gene Atlas (TCGA). A set of 6 miRNAs with correlation weights was obtained a set of mathematical operations was performed over these miRNAs for optimum classification of healthy and lung cancer individuals. DNA computing with weighted amplification is based on polymerase‐based strand displacement (PMSD) and LCHA reactions. According to the screened miRNAs and their weight information, PMSD assigns weights for different miRNA biomarkers by releasing weight DNA strands, and LCHA amplifies these weight DNA strands to achieve a detectable fluorescence signal. PMSD provides simple chemistry for mathematical operations, and LCHA provides high sensitivity for signal amplification. In the validation of 10 NSCLC synthetic samples and 10 healthy synthetic samples, the diagnostic accuracy of the probes was 100%. Molecular computing was performed for miRNA from cell lines and clinical tissue samples. Target miRNAs of 3 NSCLC cells and 1 normal cell showed correct signals in the diagnostic. In clinical sample diagnosis, the diagnostic accuracy was 92.86% for 10 cancer‐adjacent tissues and 18 NSCLC tissues, with a total sample‐to‐result time of 2.5 h. Our molecular computing with weighted amplification provides a new strategy for marker‐based molecular diagnostics.

## 2 Results and Discussion

### 2.1 miRNA Screening and Weight Assigning

**Figure 2:**
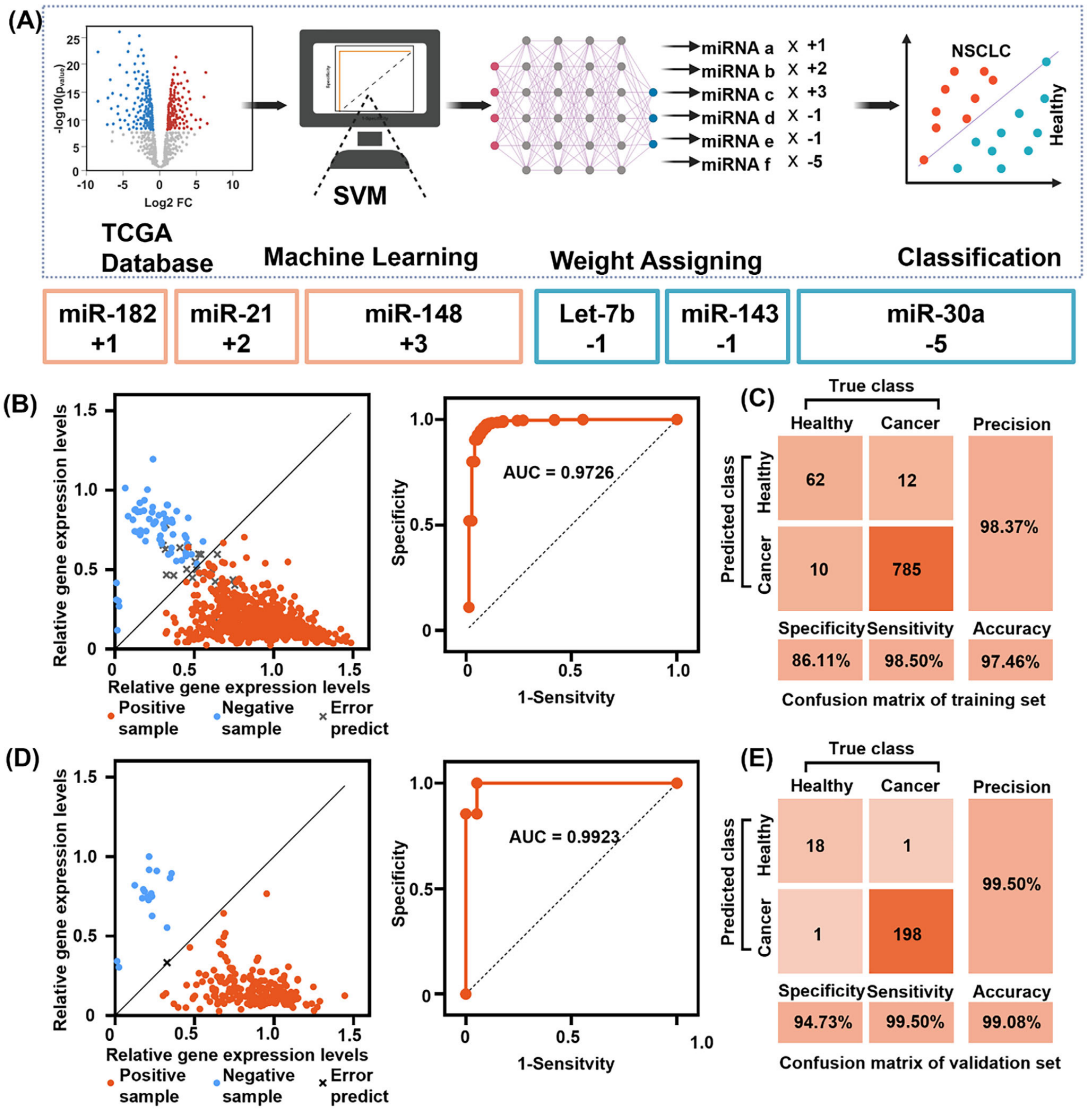
miRNA screening and weight assigning for classification performance assessment. A) Workflow of miRNA screening and weight assigning. miRNA expression profiles of publicly available healthy and NSCLC individuals from the TCGA database, candidate miRNAs were preliminarily screened, miRNA combinations were selected from candidates and assigned with weights by SVM algorithm, evaluation of classification performance. Six miRNAs and their weights were obtained. B) The screened miRNAs and their corresponding weights were used to classify the training set with good classification results, AUC = 0.97. C) Confusion matrix analysis of training set (*n* = 869), with an accuracy of 97.46%. D) The screened miRNAs and weight combinations were used to classify the validation set with good classification results, AUC = 0.99. E) Confusion matrix analysis of the validation set (*n* = 218), with an accuracy of 99.08%.

To obtain a set of miRNAs and the corresponding weights for classifying NSCLC and healthy individuals, SVM a supervised learning model with a specific algorithm was used.^[^ ^40^ ^]^ SVM is well‐suited for miRNA‐based classification tasks due to its ability to identify the optimal hyperplane for separating classes. In the context of miRNA expression data for NSCLC, which often exhibits complex and subtle patterns, SVM can effectively discriminate between NSCLC‐associated and normal miRNA profiles. Furthermore, miRNA datasets are typically high‐dimensional, where SVM tends to outperform alternatives such as neural networks, which are more prone to overfitting, and random forests, which can become computationally intensive. These advantages make SVM a robust and efficient choice for miRNA screening in NSCLC diagnostics.^[^ ^41^, ^42^ ^]^ As shown in **Figure** **2**A, we obtained the public miRNA expression profiles of 996 NSCLC and 91 healthy individuals from the TCGA database. These data were used for differential expression analysis to classify samples into cancer and healthy groups. After preliminary screening, we obtained 30 up‐regulated and 30 down‐regulated candidate miRNAs, which were stably expressed at more than 2‐fold levels in the NSCLC group than in the healthy group (Figure S1, Supporting Information). Second, we constrained the weights to integers less than 10 and the mathematical operations to addition, multiplication, and subtraction only as application constraints for the 60 miRNA inputs in order to obtain a suitable input set without affecting the classifier accuracy. In the screening of miRNA combinations and weights, we compared three groups of miRNA combinations and their weights. The combination with the best performance was miR‐182, For miR‐21, miR‐148, let‐7b, miR‐143, and miR‐30a, the corresponding positive and negative weights are +1, +2, +3, and ‐1, ‐1, ‐5 respectively (“+” refers to the up‐regulated miRNA in NSCLC samples, “‐” refers to the down‐regulated miRNA in NSCLC samples). The above‐mentioned 6 miRNAs were used as training inputs to classify the training set (797 NSCLC and 72 healthy). The area under the curve (AUC) of the receiver operating characteristic curve was 0.97, and the classification effect was good. The sensitivity of the confusion matrix was 98.50%, the specificity was 86.11% and the accuracy was 97.46% (Figure 2B,C). The validation set was classified (199 NSCLC and 19 healthy), AUC of the receiver operating characteristic curve was 0.99, indicating a good classification effect. The sensitivity of the confusion matrix was 99.50%, specificity was 94.73%, and accuracy was 99.08% (Figure 2D,E). The performance of the other two groups is shown in Figure S2A,B (Supporting Information). We also introduced artificial noise into the validation data. For example, when *σ* = 0.1, compared with the noise‐free situation, the accuracy of the classifier only decreased by 3%. This indicates that the SVM classifier optimized with hyperparameters has strong robustness against the possible types of noise in the TCGA dataset (Figure S3, Supporting Information). In comparison, if a single target was used and the weight was not considered, the diagnostic specificity for NSCLC was 0, with an accuracy of 91.28%. All targets in the combination were used but their weights were set to 1 or −1, the diagnostic performance also declined (Figure S4, Supporting Information). This highlights the necessity of multiple targets and weight assignments. Collectively, a set of 6 miRNAs were screened and their weights were assigned that could classify NSCLC and healthy individuals.

### 2.2 Molecular Computing with Weighted Amplification

**Figure 3:**
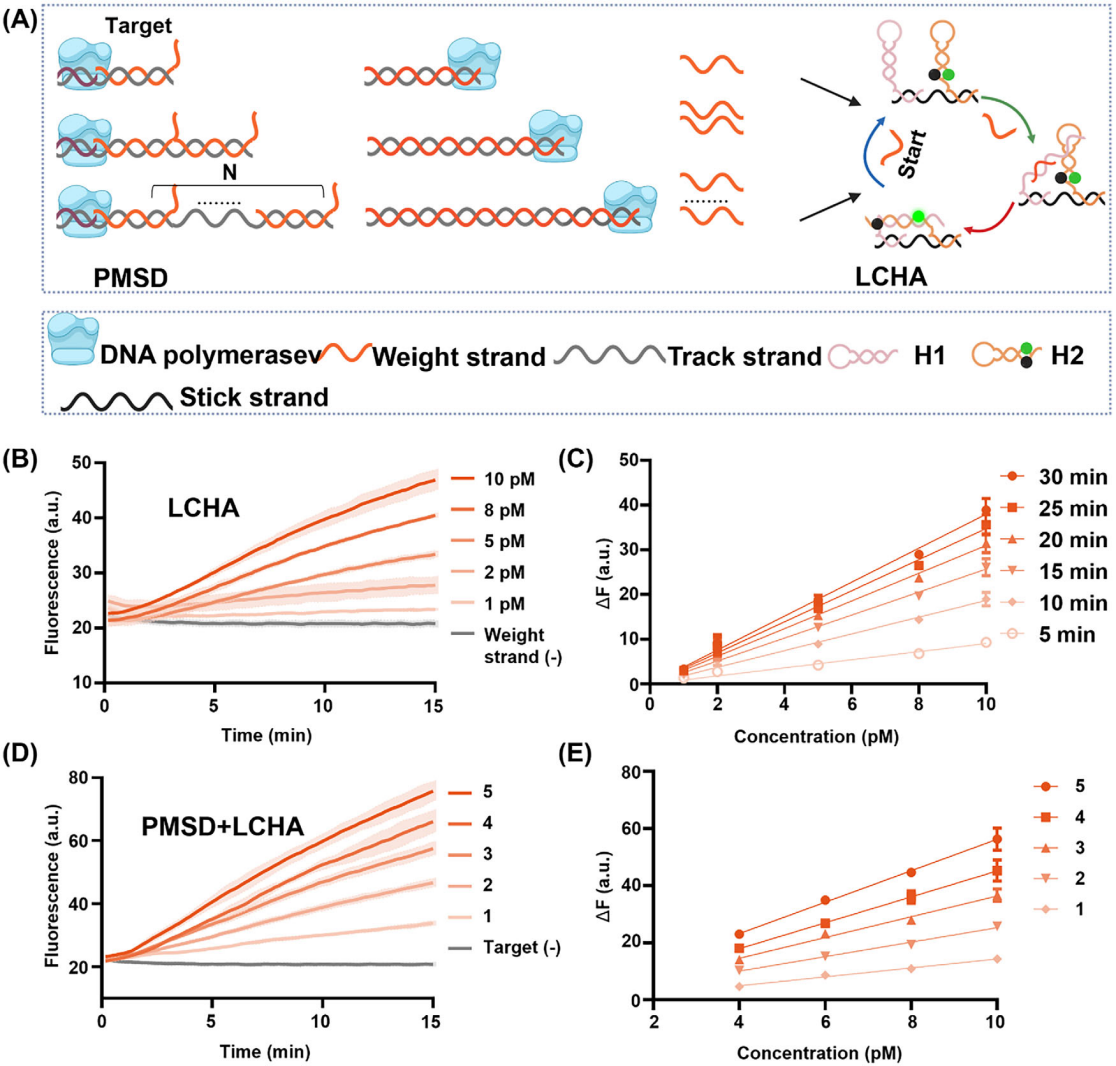
Weighted amplification molecular computing strategy construction and characterization. A) Schematic of the weighted amplification molecular computing strategy. PMSD assigns weights for different miRNA biomarkers by releasing weight DNA strands. LCHA amplifies these weight DNA strands to achieve a detectable fluorescence signal. B) Fluorescence kinetic curves generated by the LCHA were triggered by five target concentrations. C) Fluorescence‐concentration linearity every 5 min over 30 min with R^2^ > 0.98. D) Fluorescence kinetic curves generated by the weighted amplification molecular computing strategy with weights 1–5 at 10 pm. N denotes the weight of the target. E) The linear relationships of fluorescence intensity at 15 min and concentration, R^2^ > 0.96. The fluorescence‐weight linear relationship is shown in Figure S7B (Supporting Information). ΔF was calculated as the difference in fluorescence intensity between the experimental and control groups at 15 min. Condition: 10 nm PMSD, 100 nm LCHA, 3.98 nm Bst polymerase. 1×ThermoPol reaction buffer (20 mm Tris‐HCl, 10 mm (NH_4_)_2_SO_4_, 10 mm KCl, 4 mm MgSO_4_, 0.1% Triton X‐100, pH 8.8) at 25 °C. Data are mean ± S.D. (*n* = 3 independent experiments).

As shown in **Figure** **3**A, the weighted amplification molecular computing strategy contains two DNA‐based reactions. PMSD assigns weights for different miRNA biomarkers by releasing weight DNA strands. The PMSD system is assembled by mixing the track strands and weight strands in a 1:N ratio. After the targets are recognized and combined with the corresponding PMSDs, a polymerase‐mediated strand displacement reaction occurs, releasing weight strands representing target information. LCHA amplifies these weight DNA strands to achieve a detectable fluorescence signal, with hairpin 1 (H1) and hairpin 2 (H2), stick strands mixed in equal proportions. H1 is opened by the weighted strand, exposing structural domains that can be reacted with H2, and then H2 is opened as a fluorescence reporter module, and fluorescence signals are subsequently increased while the weighted strand is released into the next cycle, the more the amount of weight strands, the more LCHAs are opened at the same time. The FAM‐ and HEX‐labeled LCHAs were designed by us following the previous protocol.^[^ ^29^ ^]^ Detailed DNA sequences and modifications are shown in Table S1 (Supporting Information).

The assembly and reaction of the PMSD system were first validated by polyacrylamide gel electrophoresis (PAGE) (Figure S5A, Supporting Information), lanes 7–11 were PMSD with different weights, the higher the weight, the slower the movement, which indicated the correct assembly of PMSD. Lanes 13–17 displayed the products of the PMSD after 40 min of reaction. The extracted gray values of the weight strands in lanes 13–17 was linearly fit with weight values with R^2^ of 0.99, validating all PMSD reactions (Figure S5B, Supporting Information). Next, we performed fluorescence kinetic characterization of LCHA. As shown in Figure 3B, five target concentrations in the 1–10 pm were used to trigger the LCHA system, whose reaction rate increased as a function of concentration. Fluorescence intensities were taken out every 5 min for 30 min to fit the fluorescence‐concentration linear relationship. As shown in Figure 3C, the R^2^ at 5, 10, 15, 20, 25, and 30 min exceeded 0.97, suggesting that the LCHA achieved amplification of the different target concentrations at the above 6 time points over a 30 min range. To integrate PMSD and LCHA systems, Mg^2+^ concentration was also optimized including PMSD reaction time. The results showed that the ΔF at 4 mm concentration was 1.56 times the ΔF at 2 mm (Figure S6A,B, Supporting Information).

Finally, the weighted amplification molecular computing strategy was characterized as shown in Figure 3D and Figure S7A (Supporting Information), where the reaction rate increased with weight. All fluorescence intensities at 15 min were taken out and used to linearly fit with weight values with R^2^ > 0.98 (Figure S7B, Supporting Information). Fluorescence‐concentration linear relationships were also fitted with R^2^ > 0.96 (Figure 3E). Moreover, weight strand‐target linear relationships were also fitted with R^2^ > 0.96 (Figure S7C, Supporting Information). Given that the fluorescence intensity values were well‐suited for the addition and subtraction operations central to our design, and that this approach provided a simple and intuitive diagnostic readout, we chose to use the fluorescence intensity measured at 15 min in the kinetics curve for molecular computing. These results demonstrated that our weighted amplification molecular computing strategy has excellent weighted amplification capability.

### 2.3 Weighted Amplification Molecular Computing Strategy for miRNA

**Figure 4:**
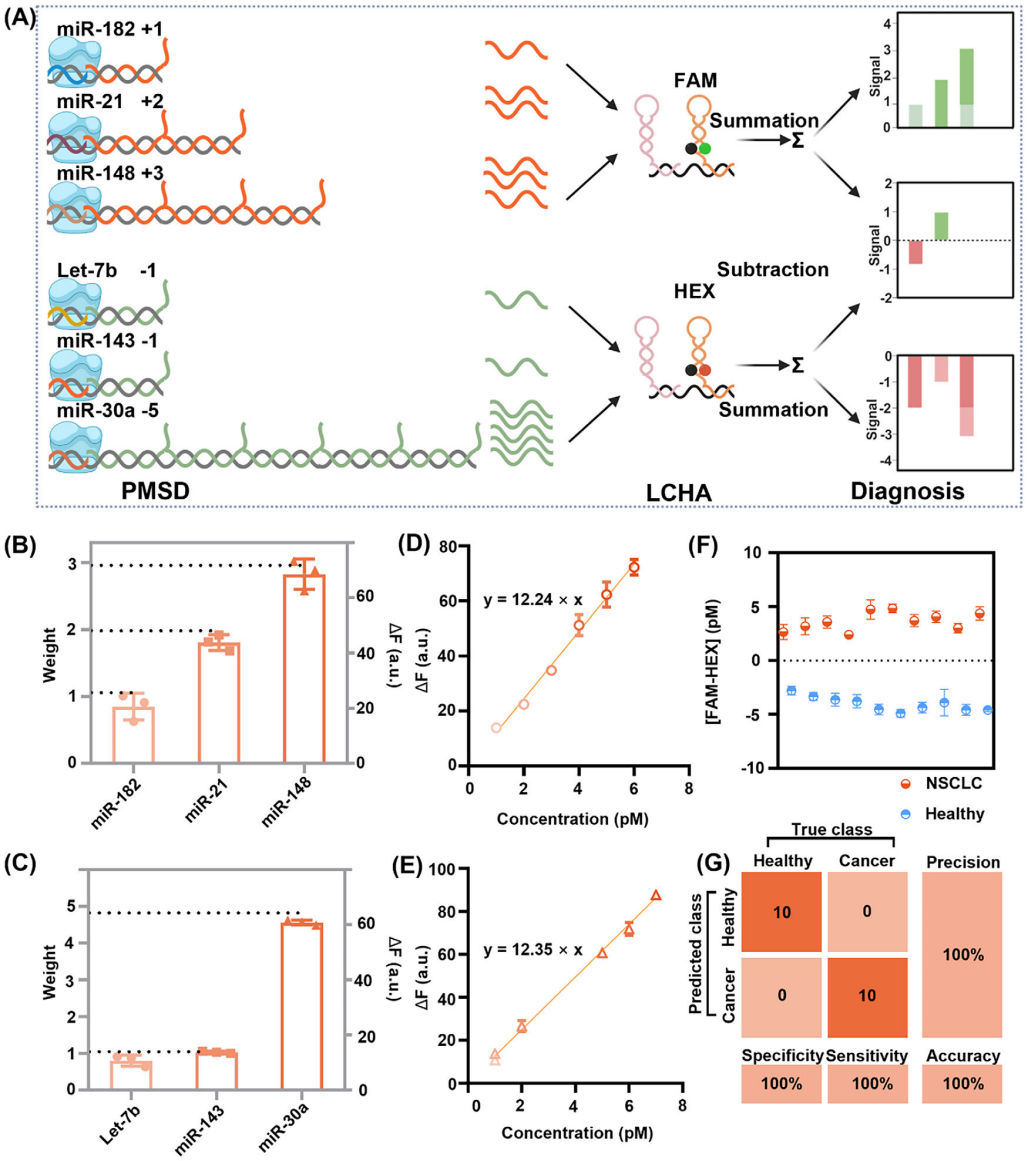
Weighted amplification molecular computing for miRNAs. A) The molecular computing workflow. Three positive and three negative weights PMSDs, each running an additive operation and an artificial subtractive operation. B) Fluorescence characterization of the three positive weights at a miRNA concentration of 10 pm. C) Fluorescence characterization of the three negative weights at an input concentration of 10 pm. The double Y‐axis shows the one‐to‐one correspondence between ΔF and weight (D) The extracted fluorescence intensities of the positive weight summation were linearly fit with weight values. E) The extracted fluorescence intensities of the negative weight summation were linearly fit with weight values. ΔF was calculated as the difference in fluorescence intensities between the experimental and control groups at 15 min. F) Diagnostic results for 10 NSCLC synthetic samples and 10 healthy synthetic samples, orange for NSCLC and blue for healthy. Data are mean ± S.D. (*n* = 3 independent experiments). G) Confusion matrix analysis of the diagnostic performance of synthetic samples. Condition: 10 nm PMSD, 100 nm LCHA, 3.98 nm Bst polymerase. 1×ThermoPol reaction buffer (20 mm Tris‐HCl, 10 mm (NH_4_)_2_SO_4_, 10 mm KCl, 4 mm MgSO_4_, 0.1% Triton X‐100, pH 8.8) at 25 °C.

As shown in **Figure** **4**A, combined with the machine learning results, we constructed three positive and three negative weights PMSDs. The fluorescence reported by the FAM‐labeled LCHA was regarded as a positive signal, and the fluorescence reported by the HEX‐labeled LCHA was regarded as a negative signal. We characterized the strategy at multiple target concentrations, and the fluorescence kinetic curves are shown in Figures S8A and S9A (Supporting Information), where the reaction rate increased with increasing weight. As shown in Figure 4B,C, the obtained fluorescence can be correlated with the target weights. Fluorescence‐concentration linear relationships were also fitted, with R^2^ exceeding 0.96 for all positive weights and 0.97 for all negative weights (Figures S8B and S9B, Supporting Information). These data showed that the weighted amplification molecular computing strategy has good molecular computing capability. Moreover, we used additive operations to obtain standard curve equations. Positive‐weight PMSDs and negative‐weight PMSDs were mixed separately for six combinations of positive‐weight targets (1, 2, 3, 1+3, 2+3, 1+2+3, Figure S10A, Supporting Information) and six combinations of negative‐weight targets (‐1, ‐1, ‐1‐1, ‐5, ‐1‐5, ‐1‐1‐5, Figure S10B, Supporting Information). The extracted fluorescence intensities of all weights were linearly fit with weight values. Positive weighted standard curve equation: *y* = 12.24 × *x* (Figure 4D), negative weighted standard curve equation: *y* = 12.35 × *x* (Figure 4E). Both equations were used as tools for sample diagnostics, and the FAM and HEX fluorescence intensities obtained from sample diagnostics were converted to [FAM] and [HEX] concentrations, respectively. When [FAM‐HEX] > 0, it indicates an NSCLC sample, and [FAM‐HEX] < 0, it indicates a healthy sample.

In order to test the diagnostic performance, we prepared synthetic samples (10 NSCLC samples and 10 healthy samples) based on miRNA expression levels in NSCLC. The miRNA combinations were shown in Tables S2 and S3 (Supporting Information), with positive weight targets highly expressed in NSCLC synthetic samples and negative weight targets highly expressed in healthy synthetic samples. Fluorescence kinetic curves were shown in Figures S11 and S12 (Supporting Information), with a significant increase in FAM fluorescence in the NSCLC synthetic sample diagnostic and a significant increase in HEX fluorescence in the healthy synthetic sample diagnostic. We used the fluorescence intensity at 15 min in the kinetics curves for result analysis. The molecular computing results are shown in Figure 4F. The confusion matrix was utilized to determine the performance of the diagnosis with 100% accuracy (Figure 4G). All results have shown that this weighted amplification molecular computing strategy has the potential to explore clinical samples.

### 2.4 NSCLC Cells and Clinical Samples Diagnosis

**Figure 5:**
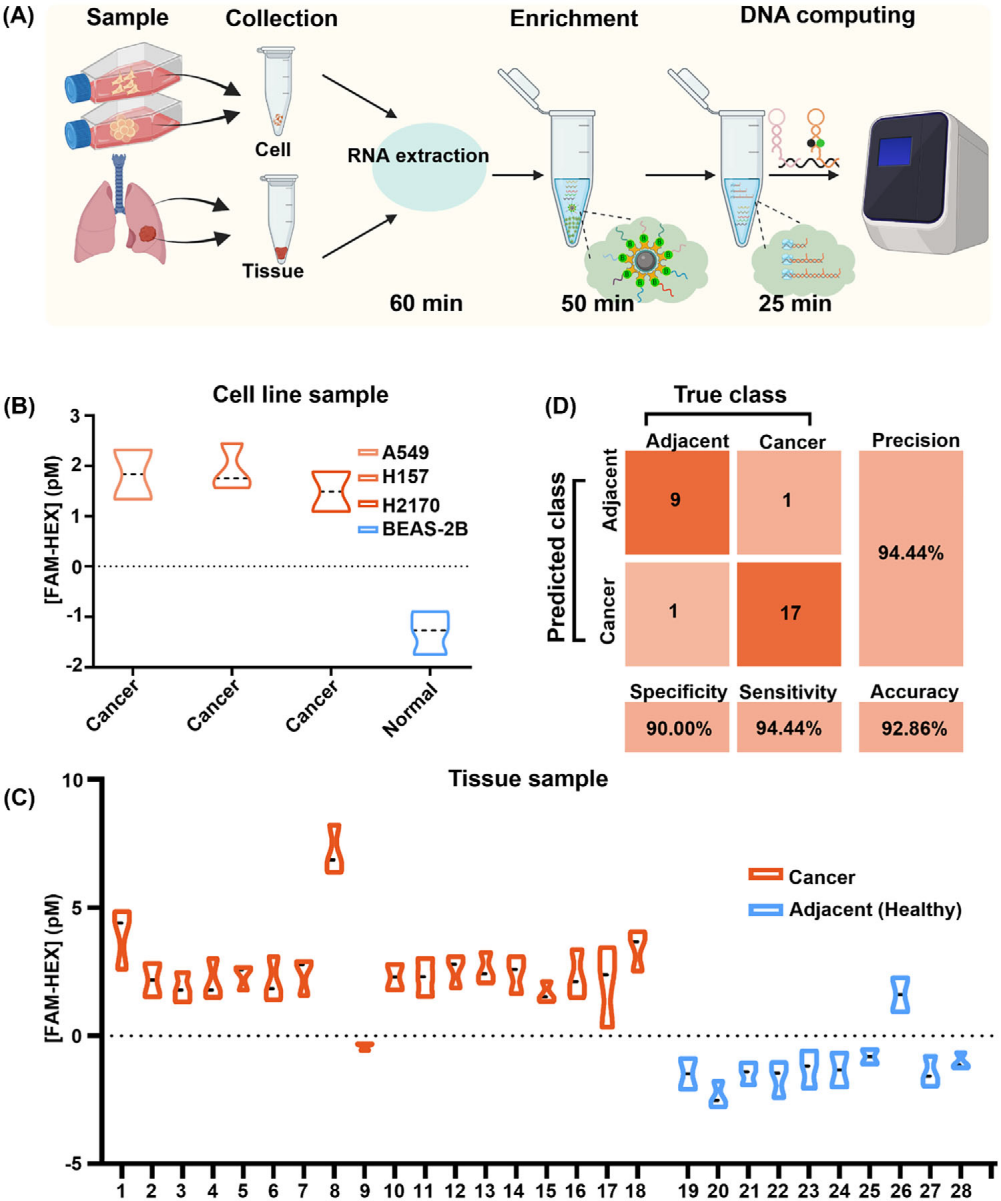
NSCLC cell line and clinical sample diagnostics. A) The entire procedure from sample to result. B) Cell line results. All NSCLC cell results [FAM‐HEX] > 0, normal cell results [FAM‐HEX] < 0. C) Diagnostic results of clinical tissues, [FAM‐HEX] > 0 indicates NSCLC, and [FAM‐HEX] < 0 indicates adjacent. D) Confusion matrix for diagnosis of 18 NSCLC samples and 10 adjacent cancer samples with 92.86% accuracy. Condition: 10 nm PMSD, 100 nm LCHA, 3.98 nm Bst polymerase. 1×ThermoPol reaction buffer (20 mm Tris‐HCl, 10 mm (NH_4_)_2_SO_4_, 10 mm KCl, 4 mm MgSO_4_, 0.1% Triton X‐100, pH 8.8) at 25 °C. Data are mean ± S.D. (*n* = 3 independent experiments).

Finally, cell lines and clinical tissues were used to validate the clinical diagnostic performance of molecular computing. Cell lines were divided into NSCLC cells (A549, H157, H2170) and normal cells (BEAS‐2B). The screened miRNA targets showed clear up‐ or down‐regulation in these cells.^[^ ^43^, ^44^ ^]^ Tissue samples were divided into two categories. I: cancer tissues from 18 NSCLC patients; II: adjacent cancer tissues from 10 NSCLC patients, and the detailed information of all tissues were shown in Tables S4 and S5 (Supporting Information). As shown in **Figure** **5**A, total miRNA was first extracted from cells or tissues. Next, the magnetic beads modified with the complementary strand of target miRNAs were used to enrich the targets, and the enrichment details are shown in Figure S13A (Supporting Information). With the optimized condition (Figure S13B–G, Supporting Information), 10‐fold enrichment can be achieved. The obtained target miRNAs were mixed with PMSD and LCHA reagents for molecular computing. Fluorescence kinetic curves of molecular computing for the cell lines were shown in Figure S14 (Supporting Information), with a stronger upward trend in FAM fluorescence for NSCLC cell lines and a stronger upward trend in HEX fluorescence for normal cell lines. As described in Figure 3D, the fluorescence intensity at 15 min of the kinetics curves was used for calculation.

The results of the four‐cell line diagnostics were correct (Figure 5B). Figure 5C shows the results of clinical tissue samples, positive and negative values of [FAM]‐[HEX] can be assigned to cancer tissue and adjacent tissue (healthy), respectively. The fluorescence kinetic curves are shown in Figures S15 and S16 (Supporting Information). Tissue diagnostic performance is shown in Figure 5D, with a diagnostic sensitivity of 94.44%, a diagnostic specificity of 90.00%, and an overall accuracy of 92.86%. From sample to result, our weighted amplification molecular computing strategy enables amplification‐free clinical tissue diagnosis in 2.5 h.

## 3 Conclusion

In conclusion, we presented a weighted amplification molecular computing strategy for miRNA‐based NSCLC direct diagnostics. A set of 6 miRNAs with correlation weights were screened from the miRNA‐seq profiles of healthy and NSCLC individuals publicly available in TCGA using a SVM algorithm. According to the screened miRNAs and their weight information, PMSD assigns weights for different miRNA biomarkers by releasing weight DNA strands, and LCHA amplifies these weight DNA strands to achieve a detectable fluorescence signal. We validated this approach using NSCLC samples. NSCLC was diagnosed with a [FAM‐HEX] signal greater than 0, while healthy samples had a signal less than 0. We achieved 100% diagnostic accuracy with 20 synthetic samples, 100% with four cell lines, and 92.86% with 18 NSCLC tissues and 10 adjacent cancer tissues, all within a total time of 2.5 h. This work can be expanded to enable the digital detection of multidimensional biomarkers, enhancing personalized disease diagnosis in point‐of‐care clinics. Although these initial results are promising, the weighted amplification molecular computing strategy requires further optimization before it can be effectively applied in clinical settings. Specifically, for blood samples with low miRNA concentrations, we plan to refine target extraction, enhance enrichment processes, and explore advanced signal amplification methods to improve sensitivity. Additionally, our strategy will need to be validated through a large‐scale clinical cohort, incorporating more detailed data, to fully assess its potential for early cancer diagnosis.

## 4 Experimental Section

### Reagents and Materials

All DNA strands used in this work were synthesized by Sangon Biotech (Shanghai) Co., Ltd., and the sequences are listed in Table S1 (Supporting Information). All modified DNA strands were purified by HPLC, while unmodified DNA strands were purified by ULTRAPAGE and PAGE. The miRcute miRNA isolation kit was purchased from Tiangen (Catalog No. DP501, Beijing, China). The dNTPs were purchased from Tiangen (Catalog No. CD117‐02, Beijing, China). The Streptavidin magnetic beads (1 µm) were purchased from Sangon Biotech (Order NO. D112005, Shanghai, China). The Thermopol buffer and *Bst* polymerase were obtained from New England Biolabs (NEB). Other chemicals used in this work were of analytical grade. DNase/RNase‐free deionized water was used in all experiments. DEPC‐water was used in the miRNA extraction experiment.

### miRNA Screening and Weight Assigning

The miRNA‐seq data of 996 NSCLC patients and 91 healthy individuals obtained from TCGA were used for training and testing of the SVM classifier, and the data were randomly divided into a training set and a validation set with an 8:2 ratio. Differential expression analysis was first used to identify the primary up and down‐regulated miRNAs in NSCLC and healthy groups. Second, a linear SVM classifier was trained on the basis of the miRNA expression data of the training set to classify cancer and healthy groups using LIBSVM^[^ ^45^, ^46^ ^]^ by MATLAB R2018a. This classifier was validated using a validation set (including 199 NSCLC and 19 healthy samples).

### Preparation and Characterization of PMSD and LCHA

PMSDs (10 nm) were prepared by mixing track strand and weight strand with a 1:N ratio in 1× ThermoPol buffer. The mixed DNA strands were annealed in a thermocycler by heating to 90 °C and then cooling to 25 °C at a rate of 2.5 °C per 1 min and stored at 4 °C for the subsequent experiments. The LCHAs (100 nm) were prepared according to the previous protocol. Native PAGE was used to characterize the PMSD assembly and reaction process. 5 µL of each sample (500 nm) was mixed with 2 µL of 6× loading buffer, and then 5 µL of the mixture was added to the 10% native PAGE for electrophoresis. The procedure was run at 120 V for 45 min at room temperature. The experiments were performed in 0.5 × TBE buffer (44.5 mm Tris, 44.5 mm Boric acid, 1 mm EDTA, pH 8.0). After 15 min of staining in 0.5 × TBE buffer containing 1 × SYBR gold (Invitrogen), the gel was photographed by the gel imaging system.

### The Procedure of miRNAs Detection by Weighted Amplification Molecular Computing

First, the target strands were added to the pre‐prepared PMSD and reacted at 37 °C for 10 min. The LCHA was then added, and the fluorescence was immediately recorded in the FAM or/and HEX channel of a real‐time PCR cycler (Rotor‐Gene Q, Qiagen, Germany) at a temperature of 25 °C, a gain of 9, and a time interval of 5 s.

### Cell Culture

All cell lines were purchased from Procell Life Science & Technology Co., Ltd. (Wuhan, China). The A549, H157, and H2170 cells were cultured in RPMI 1640 medium supplemented with 10% fetal bovine serum and 1% penicillin‐streptomycin at 37 °C in a humidified atmosphere containing 5% CO_2_. The BEAS‐2B cell was cultured in a DMEM medium under the same condition.

### miRNA Extraction

All clinical tissue samples were collected from Tianjin Chest Hospital (Tianjin, China) and China‐Japan Friendship Hospital (Beijing, China). The study was approved by the Ethics Committee at China‐Japan Friendship Hospital (Ethics number: 2021‐105‐k66). 50 mg tissue and 2 × 10^6^ cells were used for miRNA extraction. Total RNA was extracted and purified from cell lines with a miRcute miRNA isolation kit according to operating instructions. Total RNA was immediately enriched by magnetic beads. For real sample diagnostic experiments, three cancer cell lines and one normal cancer cell line, as well as ten cases of tumor tissues and ten cases of normal tissues, were prepared.

### Magnetic Bead Preparation and miRNA Enrichment

Magnetic bead preparation and miRNA enrichment were performed according to the instructions. The magnetic beads were coated with streptavidin, and the 3′ end of the complementary strands of target miRNAs were modified with biotin. After optimization, 2 mg magnetic beads were connected with 1 µm of each complementary strand. The optimal annealing temperature for the enrichment process is shown in Figure S12 (Supporting Information). The target miRNA was immediately used in fluorescence experiments.

### Code Availability

The SVM training and validation code in this study is available on https://github.com/Su‐Laboratory/SVM‐for‐NSCLC

## Conflict of Interest

The authors declare no conflict of interest.

## Supporting information



Supporting Information

## Data Availability

The data that support the findings of this study are available from the corresponding author upon reasonable request.
