# Supplementary material for: DNA Molecular Computing with Weighted Signal Amplification for Cancer miRNA Biomarker Diagnostics
Source: Adv Sci (Weinh). 2025 Apr 11;12(22):2416490. doi: 10.1002/advs.202416490 (PMC12165086; doi:10.1002/advs.202416490)
Supplement: Supplementary file 1 — Supporting Information [file ADVS-12-2416490-s001.pdf]

ADVANCED SCIENCE
Open Access

Supporting Information

for *Adv. Sci.*, DOI 10.1002/advs.202416490

DNA Molecular Computing with Weighted Signal Amplification for Cancer miRNA Biomarker Diagnostics

Hongyang Zhao, Yumin Yan, Linghao Zhang, Xin Li, Lan Jia, Liang Ma* and Xin Su**

Supporting Information

DNA Molecular Computing with Weighted Signal Amplification for Cancer miRNA Biomarker Diagnostics

Hongyang Zhao,[1] Yumin Yan,[1] Linghao Zhang,[1] Xin Li,[2] Lan Jia,[3] Liang Ma,[4]* and Xin Su[1]**

* Corresponding authors.
H.Z. and Y.Y. contributed equally.

This PDF file includes:

Supporting Table: Table S1 to Table S5

Supporting Figures: Figure S1 to Figure S1**6**

Supporting Tables

Table S1. Sequence of the oligonucleotides in this work

Name	Sequence (5'-3')
H2-FAM	GAG/iDabcyldT/CCCCCGCTCGGGCAACGGCAGCTTTTCCGA GCGGGGGAC/i6FAMdT/CAAAAGCTCAACTGTAATAAGATT AATCC-P
H1-FAM	GCAACGGCAGCTTTTGAGTCCCCCGCTCGGAAAAGCTGCC GTTGCCCGAGCGTTTTTTTTTTTTTTTTTTTTTAAGTGATCC ACCGTTAATAA
H2-HEX	GAG/iBHQ1dT/CCCCACTGTAATGAGAATGAGGTCCCTTACA GTGGGGAC/iHEXdT/CGGGACCTCAACTGTAATAAGATTAA TCC-P
H1-HEX	TGAGAATGAGGTCCCGAGTCCCCACTGTAAGGGACCTCAT TCTCATTACAGTTTTTTTTTTTTTTTTTTTTTAAGTGATCCA CCGTTAATAA
Stick strand	TTATTAACGGTGGATCACTTAGGATTAATCTTATTACAGTT G-P
Track-S1	GCTGCCGTTGCCCGAGCGACAAAGTTCTGTAGTGCACTGA
Track-S2	GCTGCCGTTGCCCGAGCGGCTGCCGTTGCCCGAGCGACAA AGTTCTGTAGTGCACTGA
Track-S3	GCTGCCGTTGCCCGAGCGGCTGCCGTTGCCCGAGCGGCTG CCGTTGCCCGAGCGACAAAGTTCTGTAGTGCACTGA
Track-S4	GCTGCCGTTGCCCGAGCGGCTGCCGTTGCCCGAGCGGCTG CCGTTGCCCGAGCGGCTGCCGTTGCCCGAGCGACAAAGTT CTGTAGTGCACTGA
Track-S5	GCTGCCGTTGCCCGAGCGGCTGCCGTTGCCCGAGCGGCTG CCGTTGCCCGAGCGGCTGCCGTTGCCCGAGCGGCTGCCGTT GCCCGAGCG ACAAAGTTCTGTAGTGCACTGA
miR-182-S1	GCTGCCGTTGCCCGAGCGAGTGTGAGTTCTACCATTGCCAA A
miR-21-S2	GCTGCCGTTGCCCGAGCGGCTGCCGTTGCCCGAGCGTCAA CATCAGTCTGATAAGCTA
Let-7b-S1	CCTCATTCTCATTACAGTAACCACACAACCTACTACCTCA

miR-143-S1	CCTCATTCTCATTACAGTGAGCTACAGTGCTTCATCTCAAAAAAAA
miR-30a-S5	CCTCATTCTCATTACAGTCCTCATTCTCATTACAGTCCTCATTCTCATTACAGTCCTCATTCTCATTACAGTCCTCATTCTCATTACAGTGCTGCAAACATCCGACTGAAAG
W1	CGCTCGGGCAACGGCAGCTTTTT
W2	ACTGTAATGAGAATGAGGTCCCT
miR-182-5p	TTTGGCAATGGTAGAACTCACACT
miR-21-5p	TAGCTTATCAGACTGATGTTGA
miR-148a-3p	TCAGTGCACTACAGAACTTTGT
Let-7b-5p	TGAGGTAGTAGGTTGTGTGGTT
miR-143-3p	TGAGATGAAGCACTGTAGCTC
miR-30a-3p	CTTTCAGTCGGATGTTTGCAGC
BIO-C-182	TTTTTTTTTTAGTGTGAGTTCTACCATTGCCAAA
BIO-C-21	TTTTTTTTTTTCAACATCAGTCTGATAAGCTA
BIO-C-148	TTTTTTTTTTACAAAGTTCTGTAGTGCACTGA
BIO-C-7b	TTTTTTTTTTAACCACACAACCTACTACCTCA
BIO-C-143	TTTTTTTTTTGAGCTACAGTGCTTCATCTCA
BIO-C-30a	TTTTTTTTTTGCTGCAAACATCCGACTGAAAG

Table S2. NSCLC synthetic sample

**Sample**	**miR-182 (pM)**	**miR-21 (pM)**	**miR-148a (pM)**	**Let-7b (pM)**	**miR-143 (pM)**	**miR-30a (pM)**
S1	5	4	10	1	1	1
S2	5	5	8	1	1	1
S3	10	6	8	1	1	1
S4	6	8	6	1	1	1
S5	6	6	10	1	1	1
S6	7	10	9	1	1	1
S7	7	8	8	1	1	1
S8	8	5	10	1	1	1
S9	8	6	7	1	1	1
S10	10	9	7	1	1	1

"x pM" represents the final concentration

Table S3. Healthy synthetic sample

**Sample**	**miR-182 (pM)**	**miR-21 (pM)**	**miR-148a (pM)**	**Let-7b (pM)**	**miR-143 (pM)**	**miR-30a (pM)**
S11	1	1	1	6	2	10
S12	1	1	1	6	5	8
S13	1	1	1	4	4	6
S14	1	1	1	5	6	6
S15	1	1	1	5	5	8
S16	1	1	1	7	8	10
S17	1	1	1	7	8	9
S18	1	1	1	8	10	6
S19	1	1	1	8	8	7
S20	1	1	1	9	10	7

"x pM" represents the final concentration

Table S4. Summary of the cancer tissue sample information

Number	Gender	Age(year)	Caner type	Stage (TNM)
1	female	58	AD	IA
2	male	62	AD	IA
3	male	70	SC	IB
4	male	79	SC	IIB
5	male	73	AD	IIIA
6	male	66	SC	I
7	male	70	SC	II
8	male	49	SC	II
9	male	74	SC	IIIA
10	male	61	SC	III
11	female	57	SC	III
12	female	58	AD	IIB
13	female	80	AD	I
14	female	64	AD	I
15	male	61	AD	II
16	male	59	SC	II

17	male	74	AD	II
18	male	52	SC	III

AD = adenocarcinoma, SC = squamous cell carcinoma

Table S5. Summary of the adjacent cancer tissue sample information

Number	Gender	Age(year)
19	female	58
20	male	62
21	male	70
22	male	79
23	male	73
24	male	54
25	male	71
26	male	46
27	female	61
28	female	64

Supporting Figures

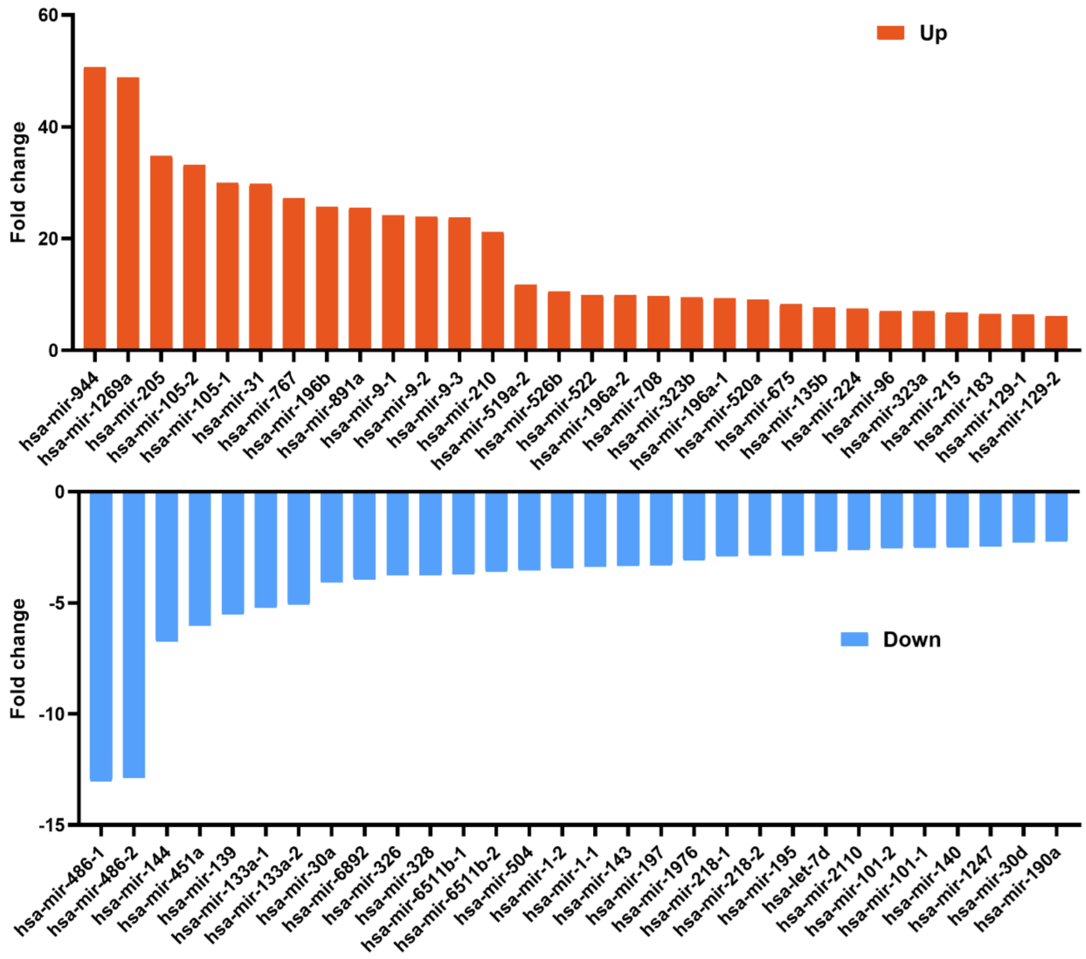

Figure S1. miRNAs expression fold changes (>2) in the NSCLC samples compared to those of healthy samples. The original data were obtained from TCGA database.

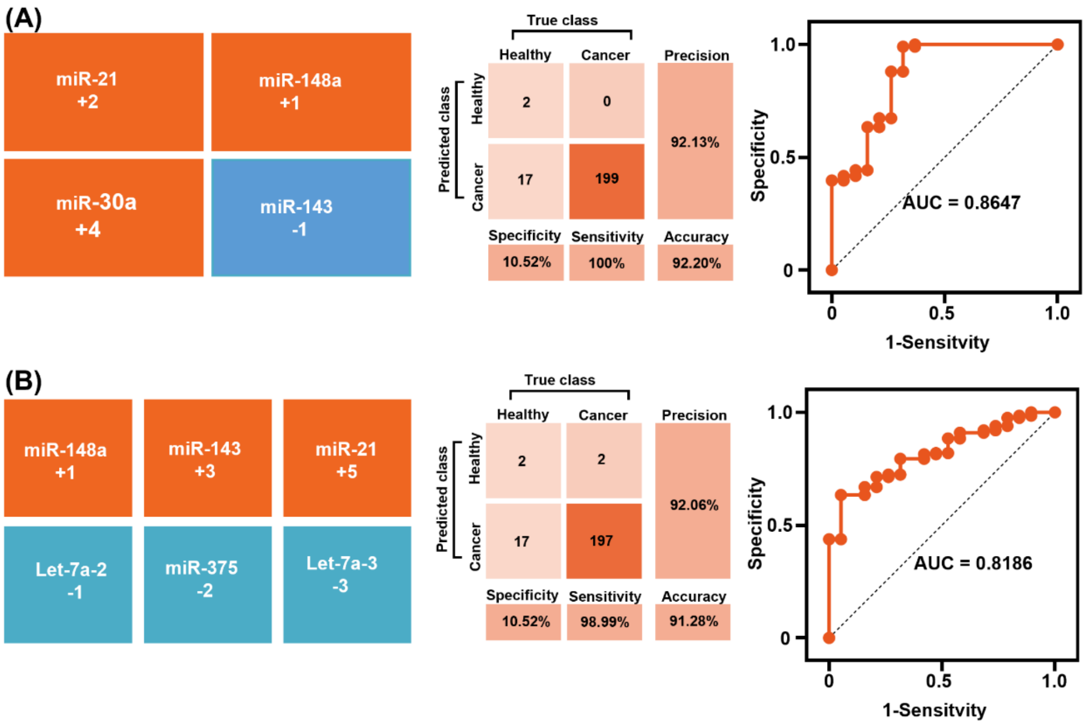

Figure S2. Different miRNA combinations and weights obtained by SVM. (A) Model with the combination of miR-21, miR-148a, miR-30a, miR-143 and the corresponding weights. Confusion matrix analysis of 218 samples with the sensitivity of 100% and specificity of 10.52%. The resulted recognition AUC of 0.8647 was obtained. (B) Model with the combination of miR-148a, miR-143, miR-21, Let-7a-2, miR-375, Let-7a-3 and the corresponding weights. Confusion matrix analysis of 218 samples with the sensitivity of 98.99% and specificity of 10.52%. The resulted recognition AUC of 0.8186 was obtained.

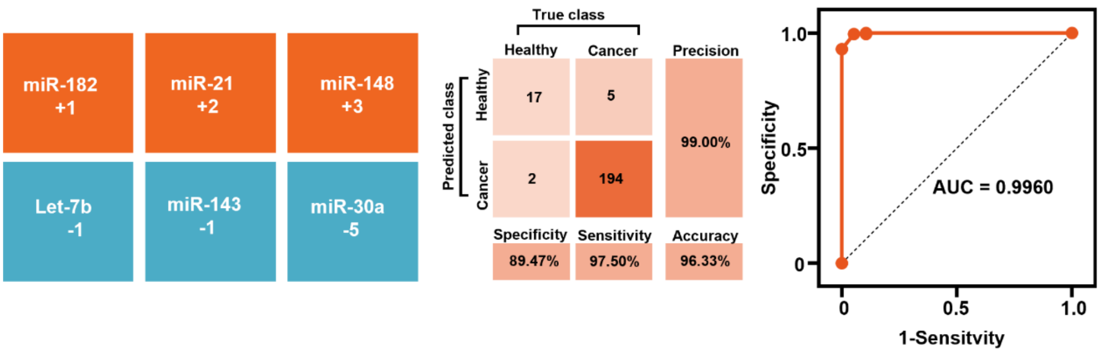

Figure S3. The impact of artificial noise on classification performance. Artificial noise with $\sigma = 0.1$ was added to the validation set, and the accuracy rate during the division phase decreased by only 3%.

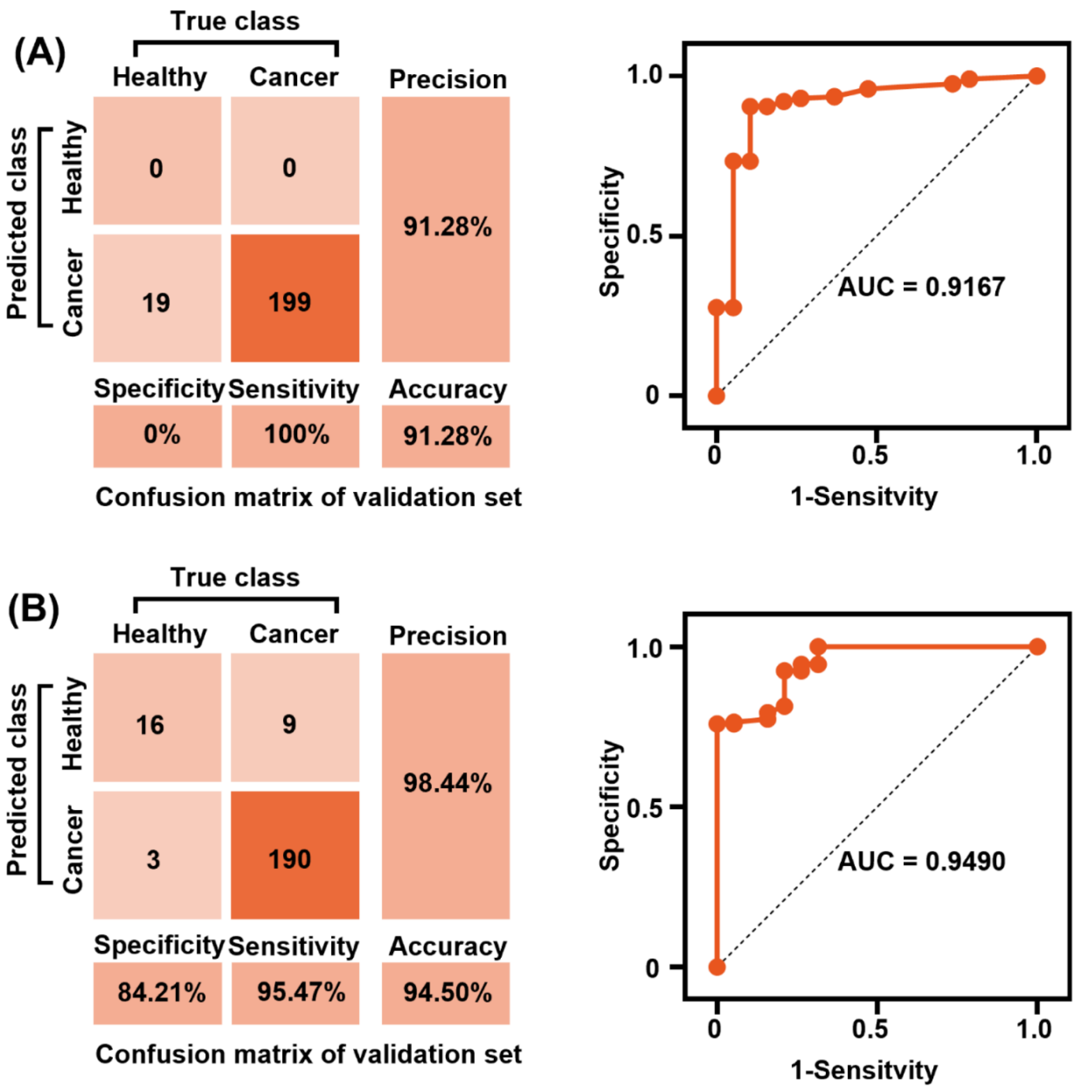

Figure S4. (A) The diagnostic performance of single miRNA (B) The diagnostic performance of the same miRNA combination as in Figure 2A. But all the weight was set to 1 or -1. The data were processed based on the same 218 randomly selected samples from the TCGA database. SVM algorithm was used.

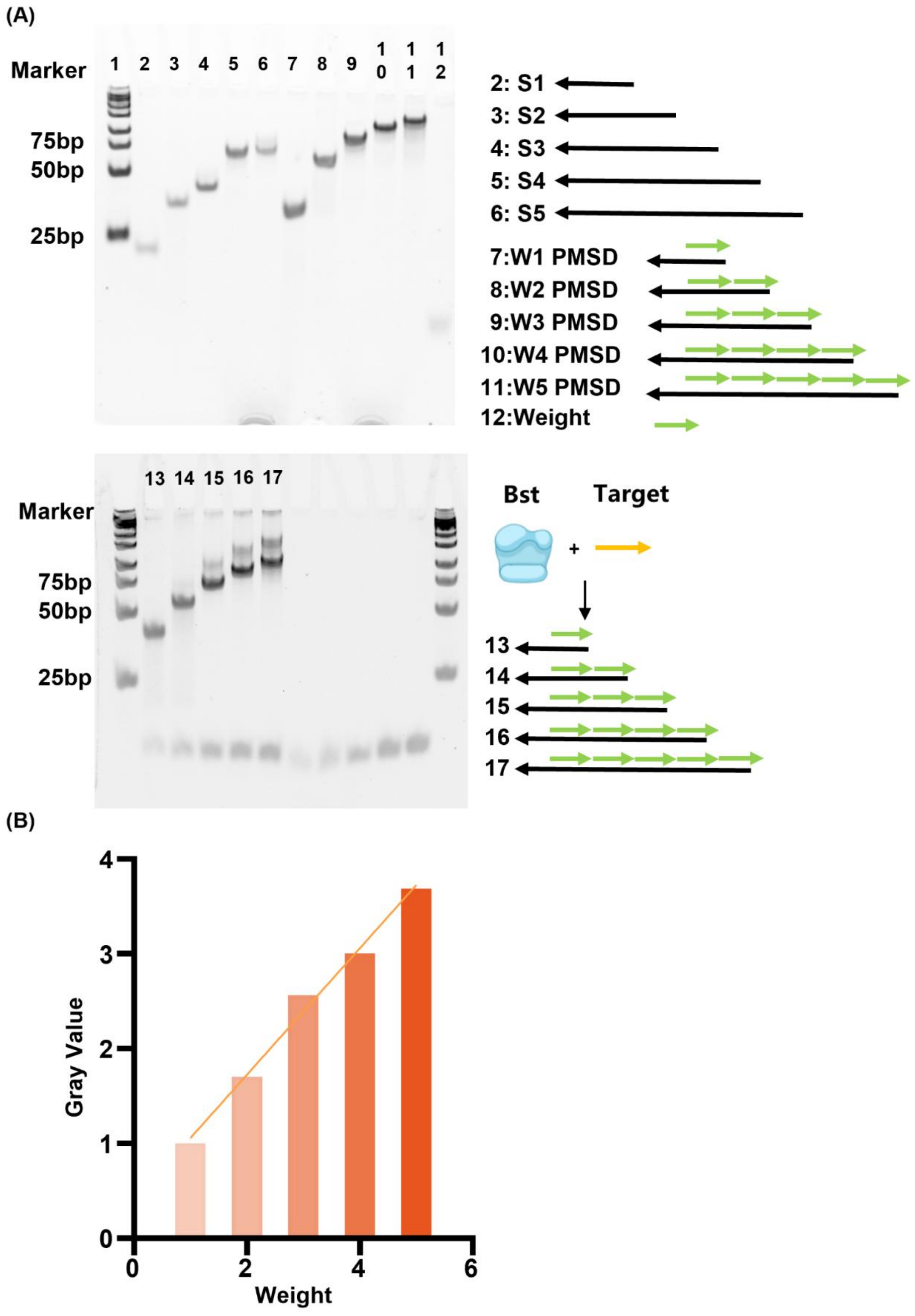

Figure S5. Gel electrophoresis verified the synthesis and reaction of PMSD. (A) Synthetic characterization. Lane 1: marker; Lane 2-6: 500 nM Track S1-S5; Lane 7-11: 500 nM PMSD. (B) Reaction characterization. Lane 13-17: 500 nM PMSD + Target + *Bst* polymerase. The extracted gray values of the weight strands in lanes 13-17 were linearly fit with weight values.

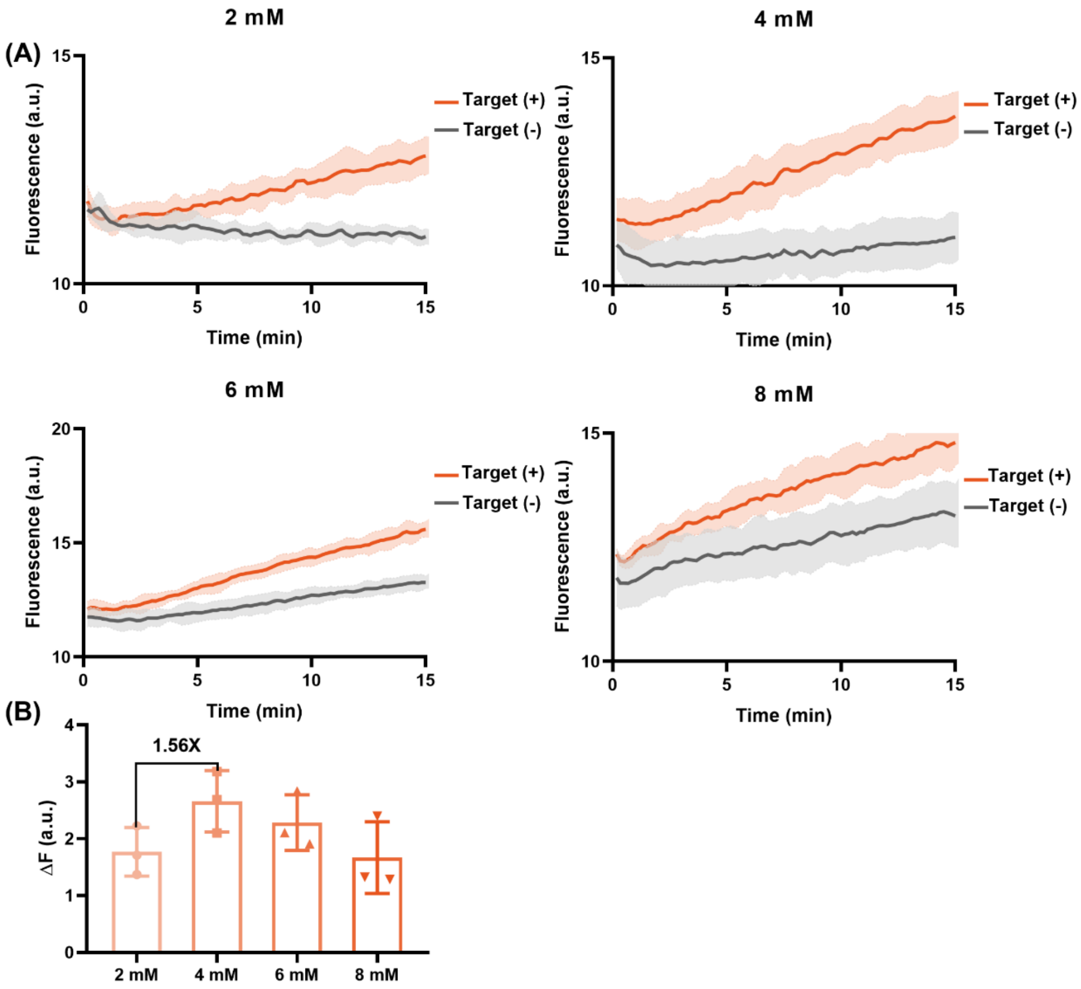

Figure S6. Mg^{2+} concentration optimization. (A) Fluorescence kinetic curves of reactions with different Mg^{2+} concentrations. (B) Fluorescence values of four Mg^{2+} concentrations at 15 min, 4 mM was the optimal concentration. ΔF was calculated as the difference in fluorescence values between the experimental and control groups at 15 min. Condition: 10 nM PMSD and miR-148, 100 nM LCHA, 3.98 nM *Bst* polymerase. Temperature is 25 °C and buffer is Thermol pol buffer. Data are mean ± S.D. (n = 3 independent experiments).

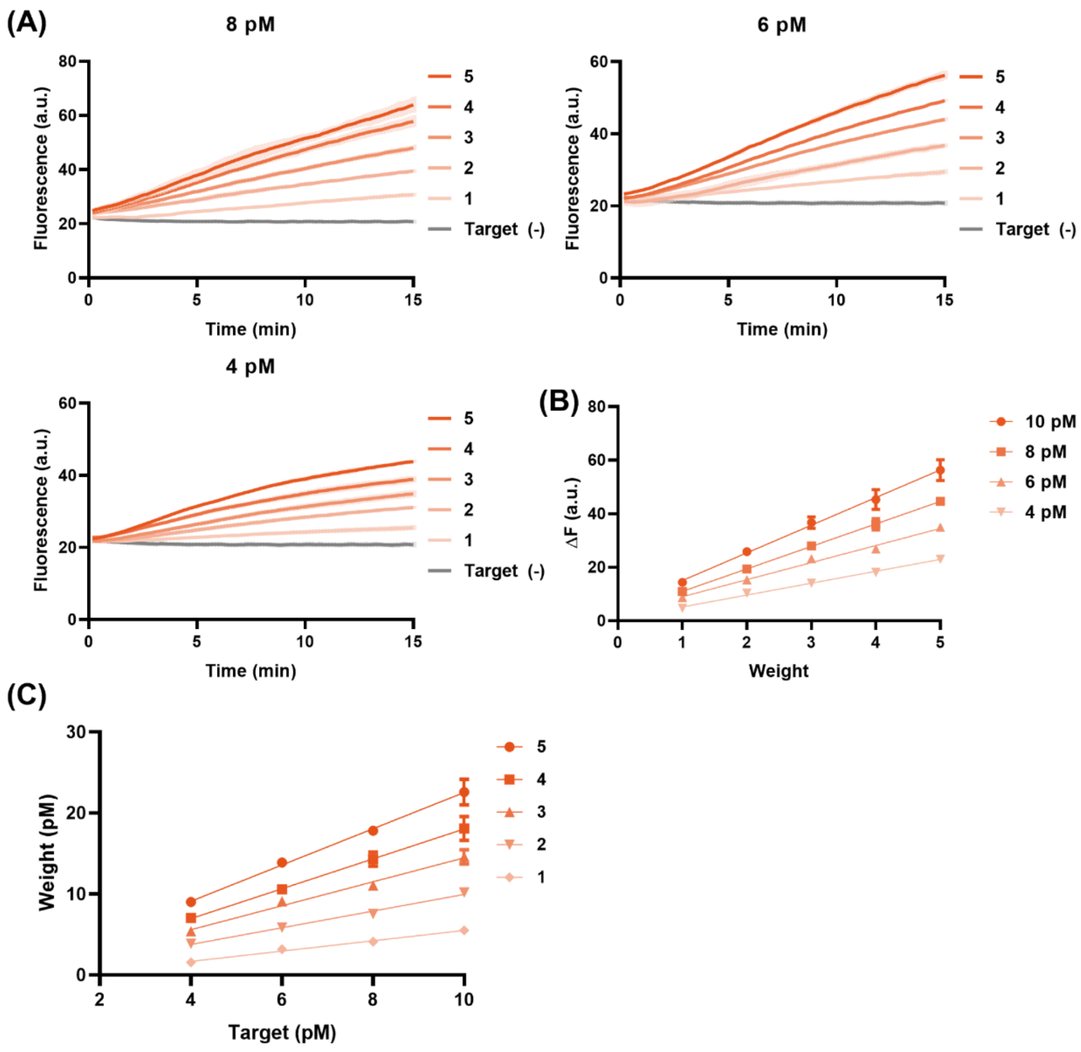

Figure S7. Signals of varied target concentrations. (A) Fluorescence kinetics curves of 8, 6, and 4 pM targets. N denotes the weights of the targets. (B) ΔF-weight linear relationship, $R^2 > 0.96$. ΔF was calculated as the difference in fluorescence values between the experimental and control groups at 15 min. (C) Linear relationship of weight strand and target in PMSD-LCHA system, $R^2 > 0.96$. Condition: 10 nM PMSD, 100 nM LCHA, 3.98 nM *Bst* polymerase. Temperature is 25 °C and buffer is Thermol pol buffer. Data are mean ± S.D. (n = 3 independent experiments).

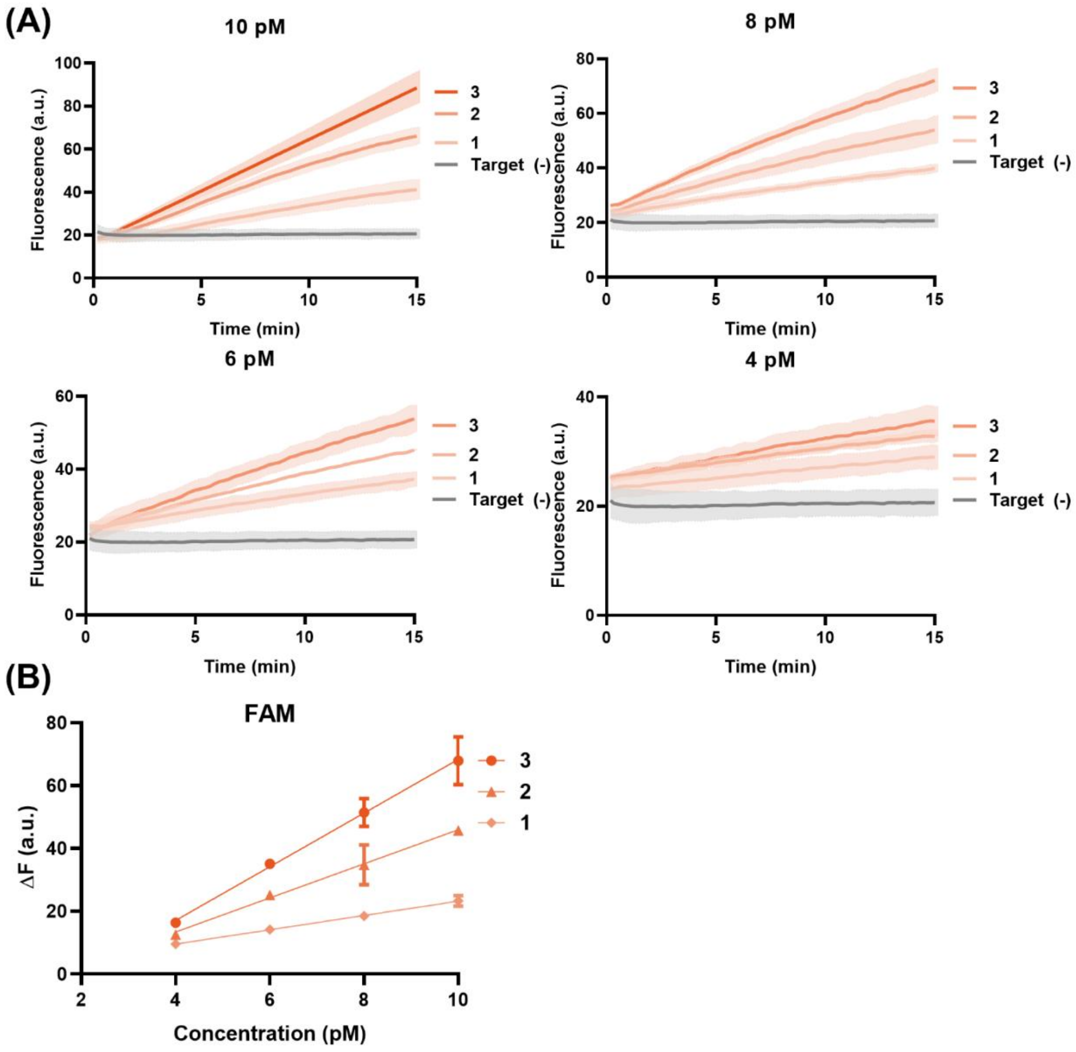

Figure S8. Testing of molecular computing capability by varied target concentrations. (A) Fluorescence kinetics curves of targets with positive weights at 10, 8, 6, and 4 pM. N denotes the weights of the targets. (B) ΔF- concentration linear relationship, R^2 > 0.96. ΔF was calculated as the difference in fluorescence values between the experimental and control groups at 15 min. Condition: 10 nM PMSD and, 100 nM LCHA, 3.98 nM *Bst* polymerase. Temperature is 25 °C and buffer is Thermol pol buffer. Data are mean ± S.D. (n = 3 independent experiments).

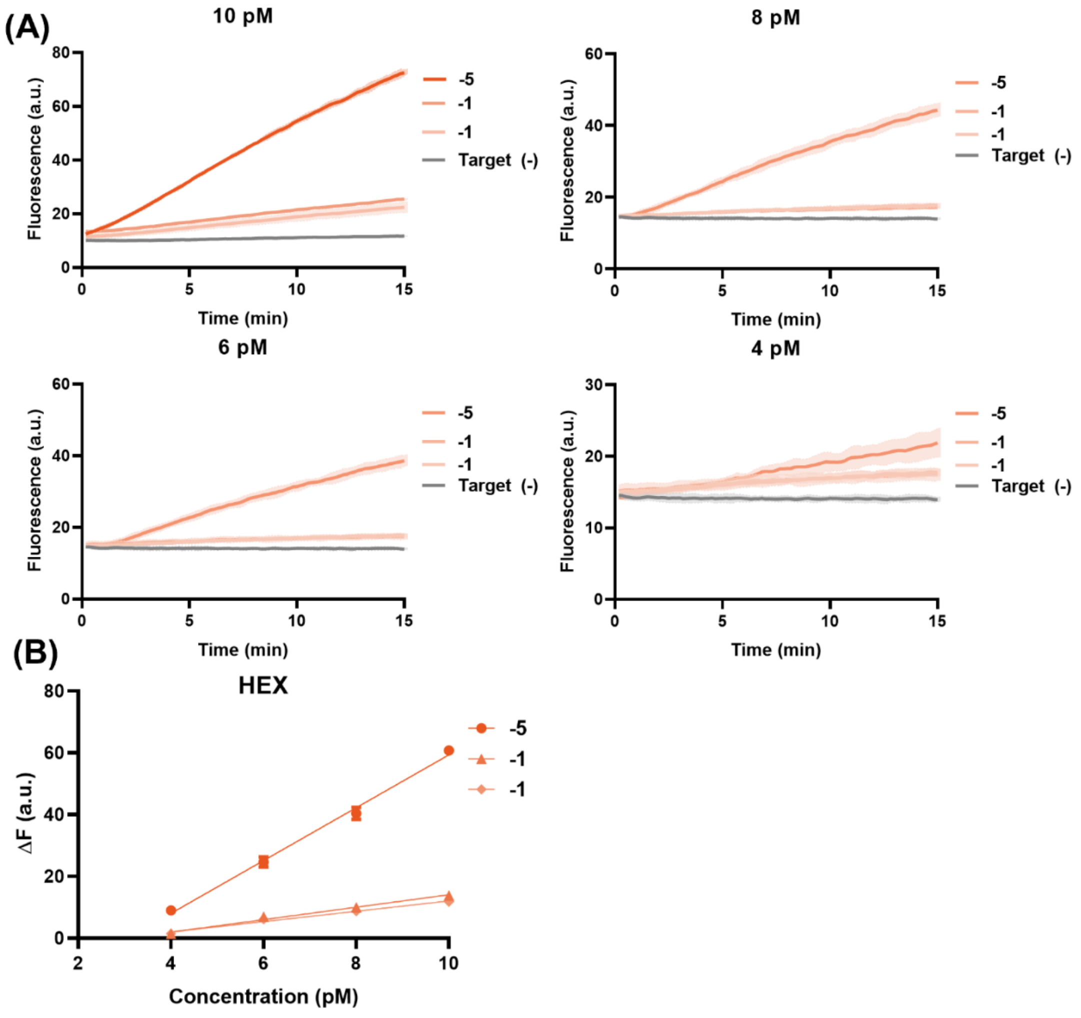

Figure S9. Testing of molecular computing capability by varied target concentrations. (A) Fluorescence kinetics curves of targets with negative weights at 10, 8, 6, and 4 pM. N denotes the weights of the targets. (B) ΔF- concentration linear relationship, $R^2 > 0.97$. ΔF was calculated as the difference in fluorescence values between the experimental and control groups at 15 min. Condition: 10 nM PMSD and, 100 nM LCHA, 3.98 nM *Bst* polymerase. Temperature is 25 °C and buffer is Thermol pol buffer. Data are mean ± S.D. (n = 3 independent experiments).

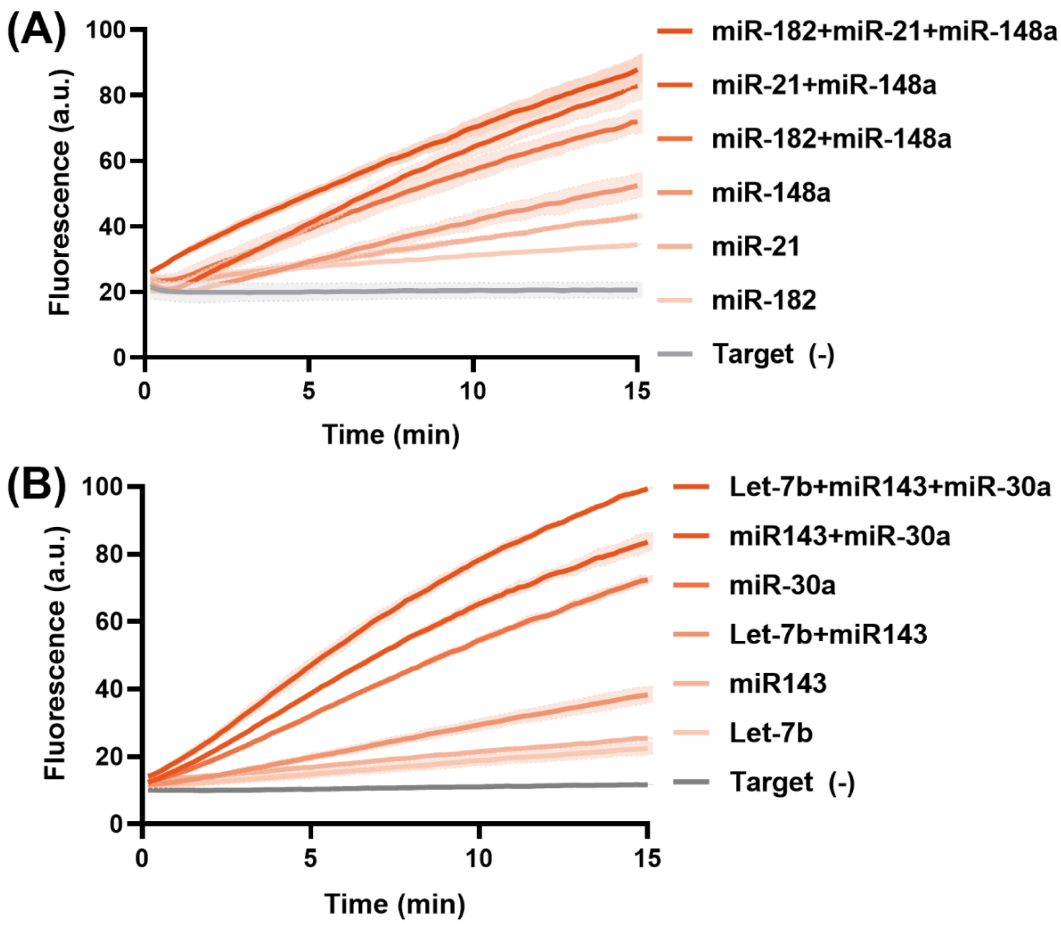

Figure S10. Signals of the summation operation of computing. (A) Fluorescence kinetics curves of targets with positive weights. (B) Fluorescence kinetics curves of targets with negative weights. Condition: 10 nM PMSD and, 100 nM LCHA, 3.98 nM *Bst* polymerase. Temperature is 25 °C and buffer is Thermol pol buffer. Data are mean ± S.D. (n = 3 independent experiments).

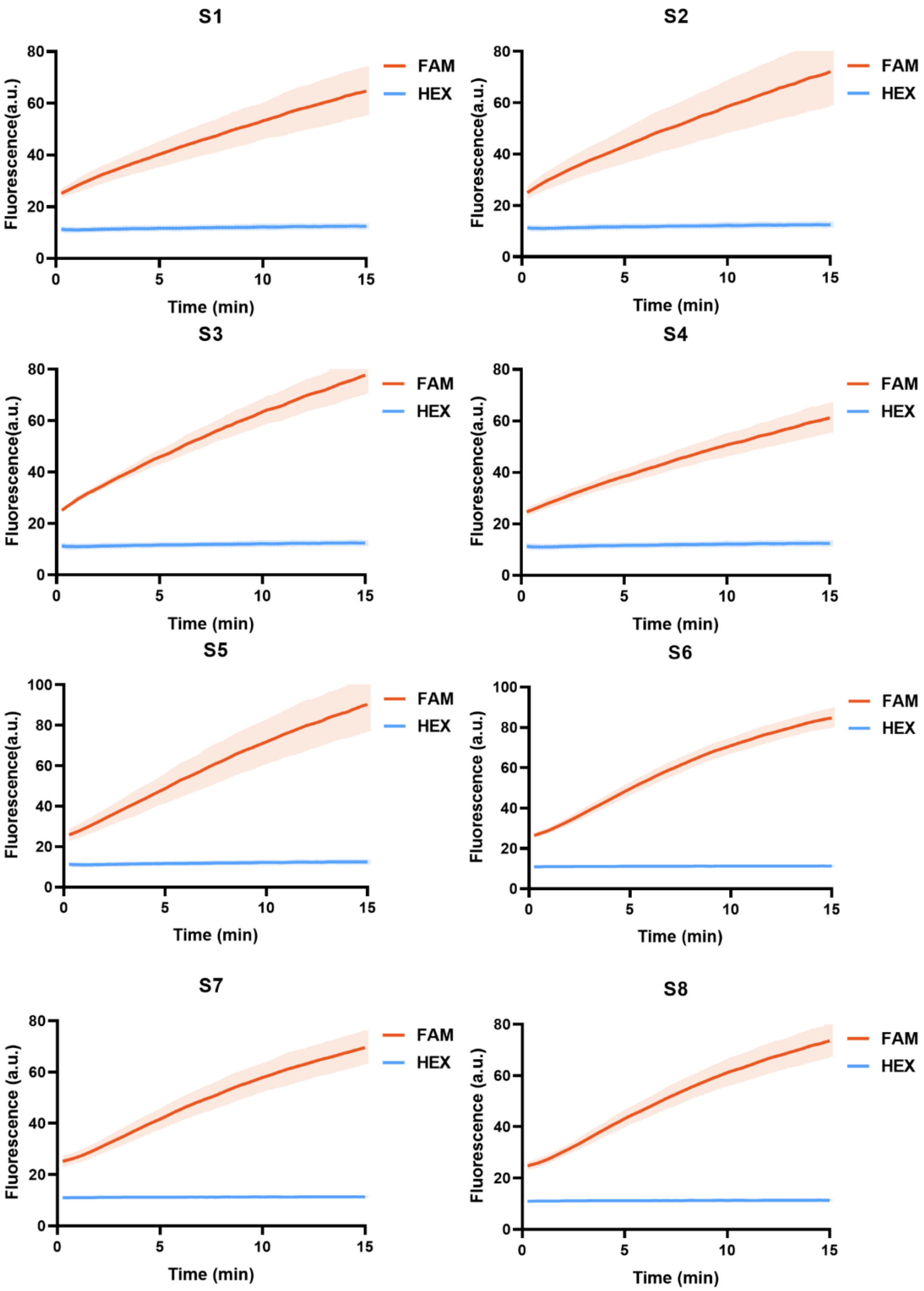
S1
S2
S3
S4
S5
S6
S7
S8
FAM
HEX
Fluorescence(a.u.)
Fluorescence (a.u.)
Time (min)
0
5
10
15
20
40
60
80
100

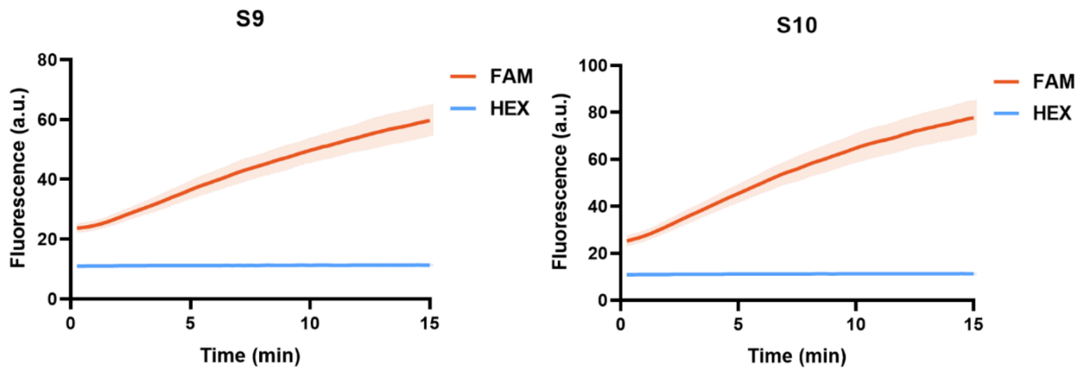

Figure S11. Fluorescence kinetic curves of synthetic NSCLC samples (for details see Table S2). Condition: 10 nM PMSD and, 100 nM LCHA, 3.98 nM *Bst* polymerase. Temperature is 25 °C and buffer is Thermol pol buffer. Data are mean ± S.D. (n = 3 independent experiments).

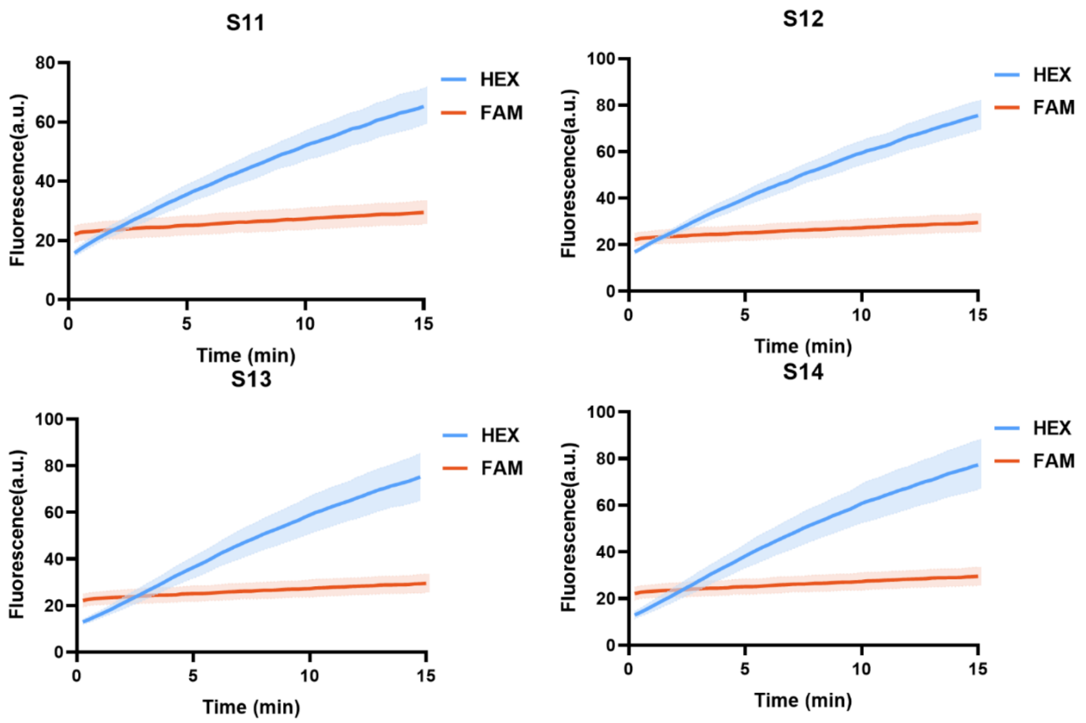

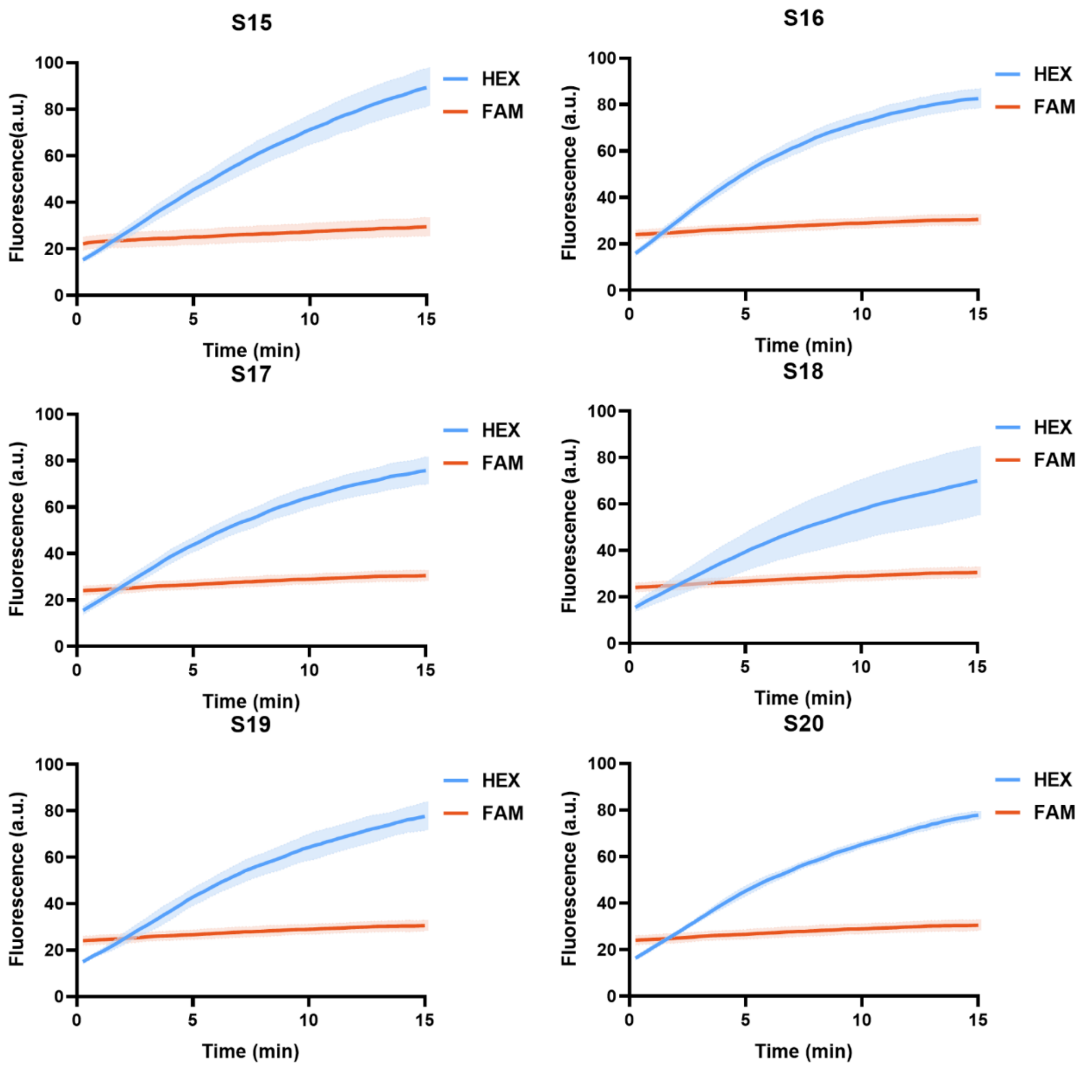

Figure S12. Fluorescence kinetic curves of synthetic health samples (for details see Table S2). Condition: 10 nM PMSD and, 100 nM LCHA, 3.98 nM *Bst* polymerase. Temperature is 25 °C and buffer is Thermol pol buffer. Data are mean ± S.D. (n = 3 independent experiments).

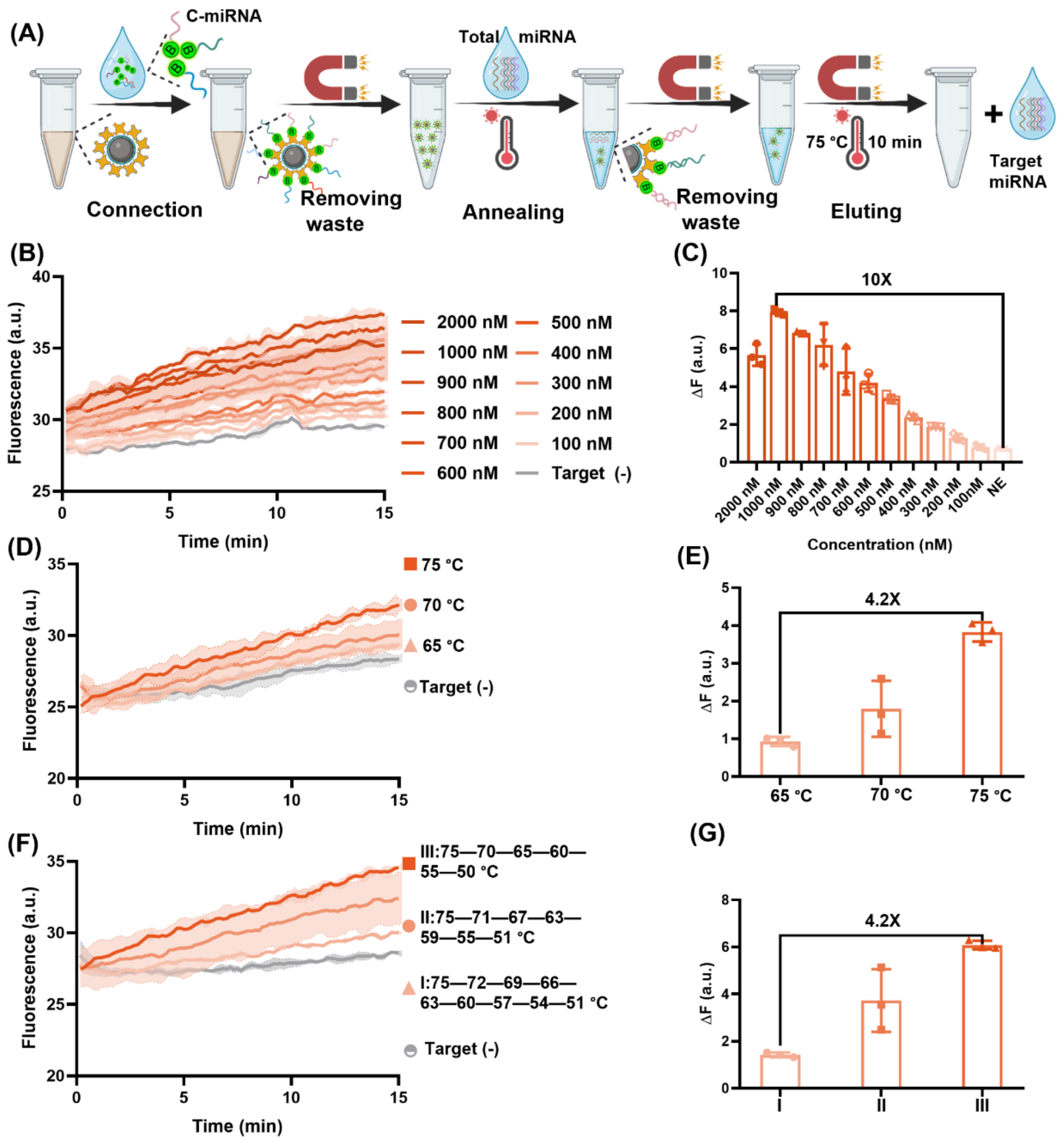

Figure S13. (A) Workflow of the miRNA enrichment: 1) modifying magnetic beads with the complementary strand of target miRNAs by biotin-streptavidin interaction; 2) removing excess complementary strands; 3) mixing and annealing of total miRNAs and magnetic beads; 4) removing non-target miRNAs; 5) eluting the miRNA by heating. (B) Fluorescence kinetics curves of molecular computing of miR-148 by using 100 to 2000 nM complementary strands. NE denotes without enrichment. (C) ΔF at 15 min of panel B. 1000 nM was the optimal concentration. (D) Fluorescence kinetics curves of molecular computing of miR-148 with different maximum annealing temperatures. (E) ΔF at 15 min of panel D. (F) Fluorescence kinetics curves of molecular computing of miR-148 by different annealing procedures. I: each temperature value is maintained for 5 min; II: each temperature value is maintained for 4 min; III: each temperature value is maintained for 3 min. (G) ΔF at 15 min of panel F. ΔF was calculated as the difference in fluorescence values between the experimental and control

groups at 15 min. The condition of molecular computing is the same as Figure 4. Data are mean ± S.D. (n = 3 independent experiments).

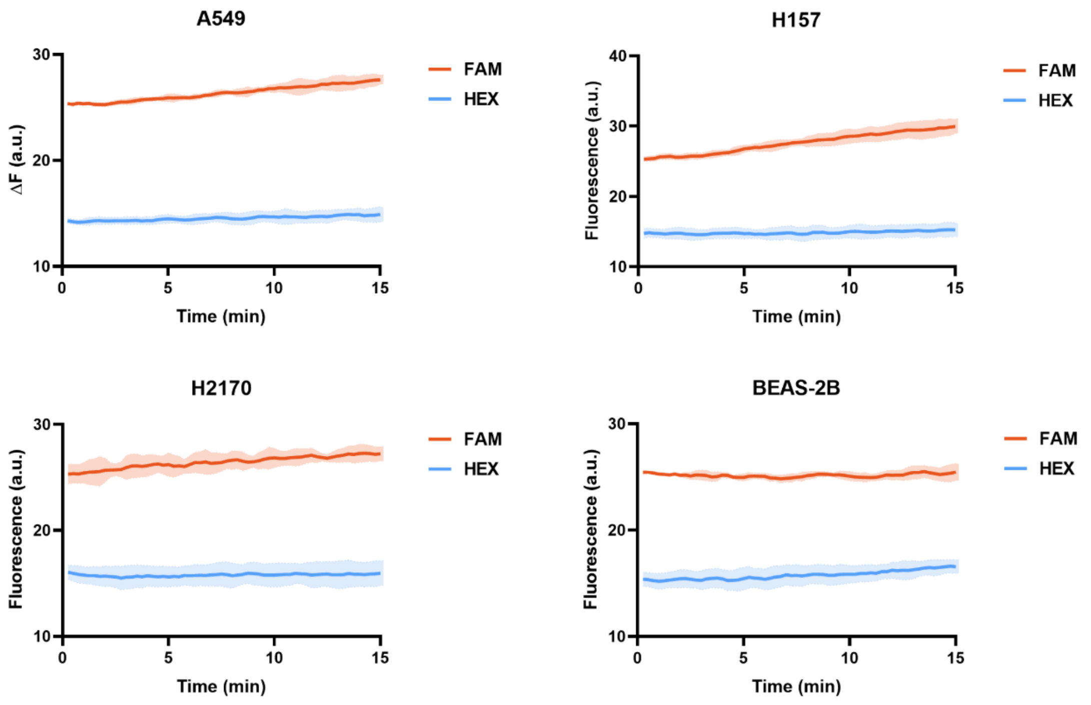

Figure S14. Fluorescence kinetics curves of the extracts of different cell lines. Condition: 10 nM PMSD and, 100 nM LCHA, 3.98 nM *Bst* polymerase. Temperature is 25 °C and buffer is Thermol pol buffer. Data are mean ± S.D. (n = 3 independent experiments).

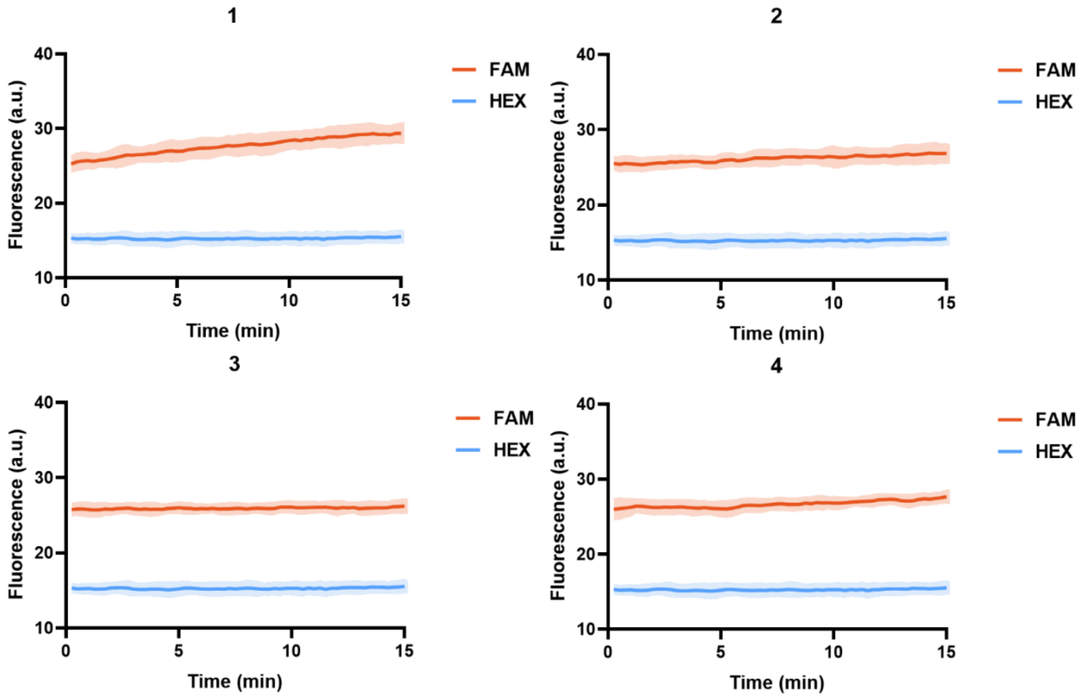

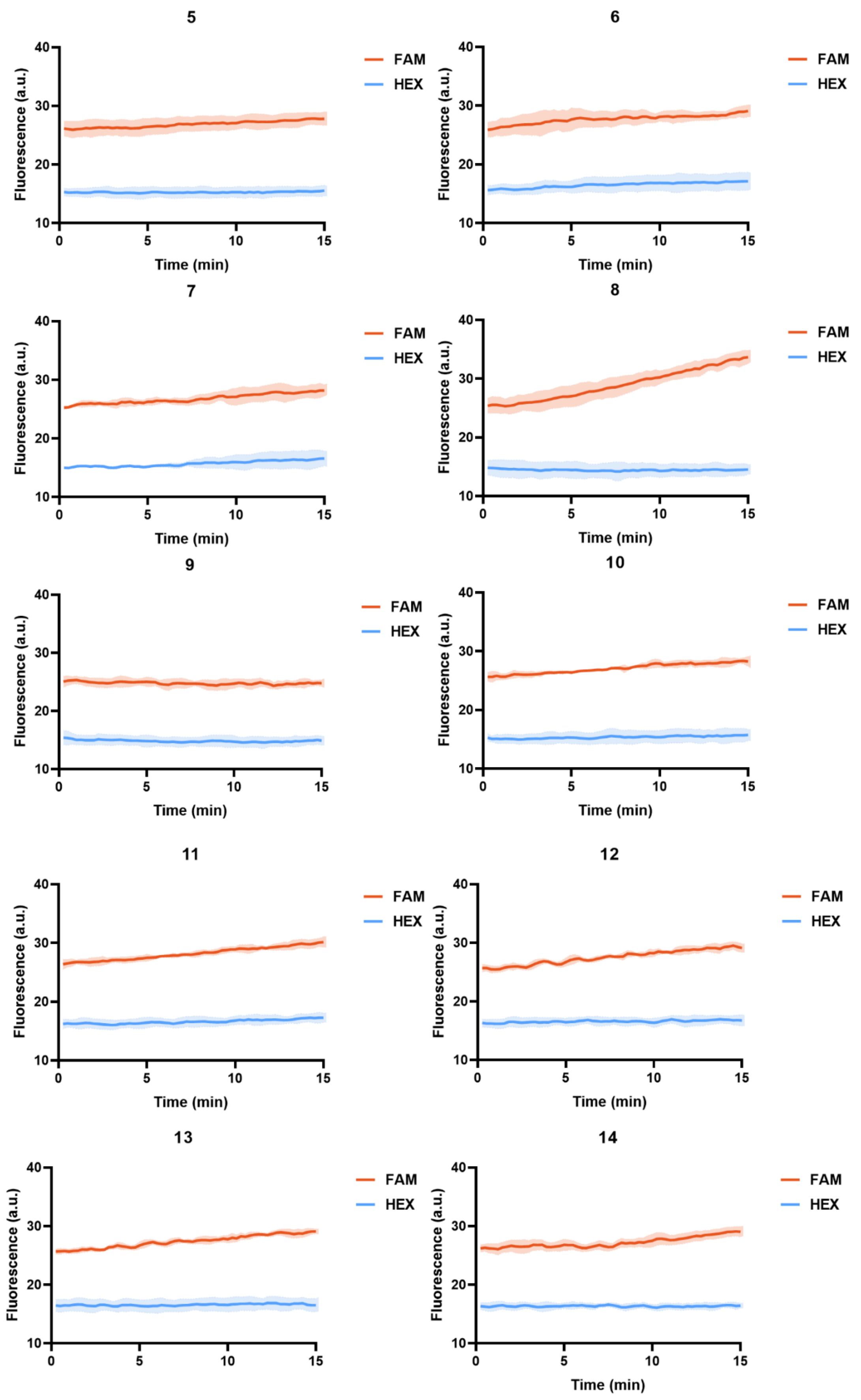
5
6
7
8
9
10
11
12
13
14
FAM
HEX
Fluorescence (a.u.)
Time (min)
10
20
30
40
0
5
10
15

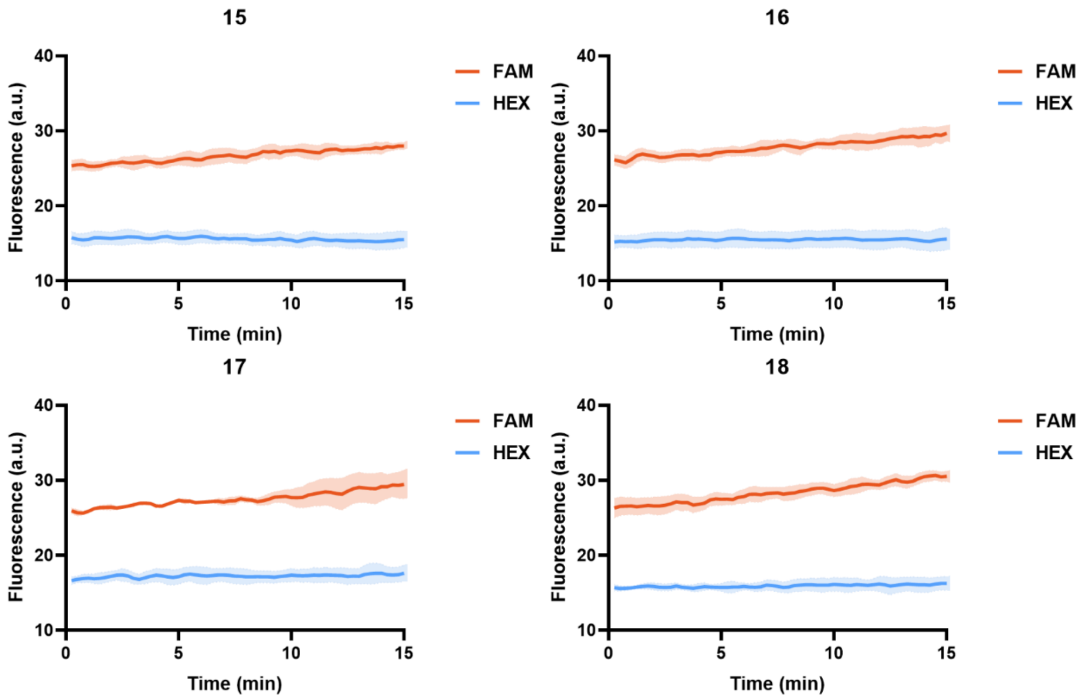

Figure S15. Fluorescence kinetics curves of NSCLC cancer tissue samples. Condition: 10 nM PMSD and, 100 nM LCHA, 3.98 nM *Bst* polymerase. Temperature is 25 °C and buffer is Thermol pol buffer. Data are mean ± S.D. (n = 3 independent experiments).

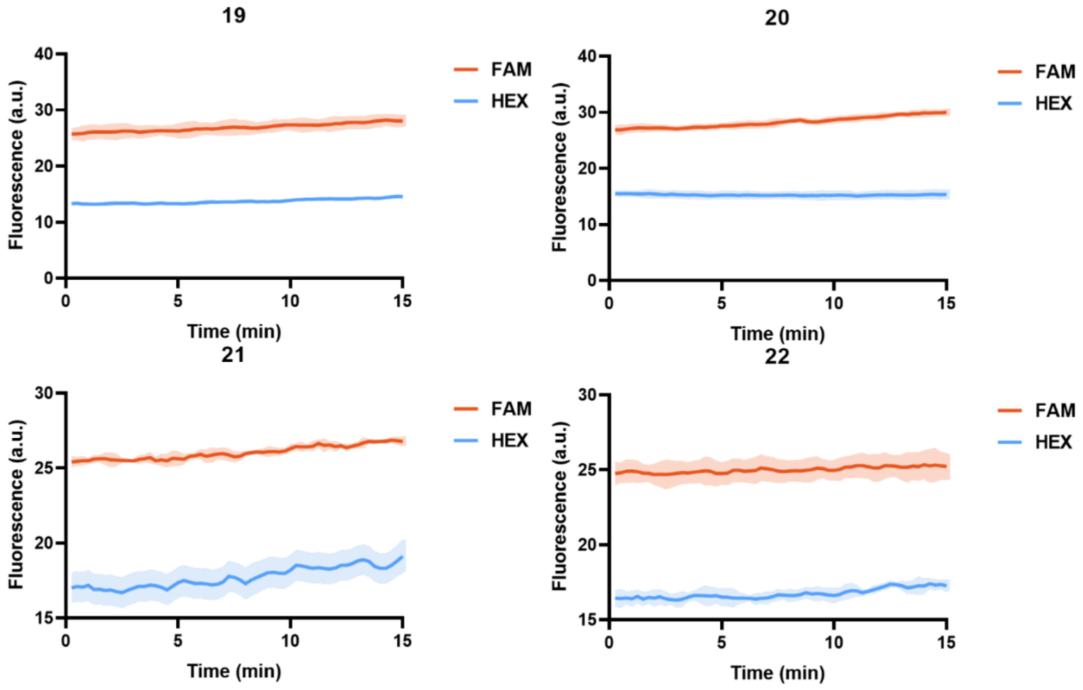

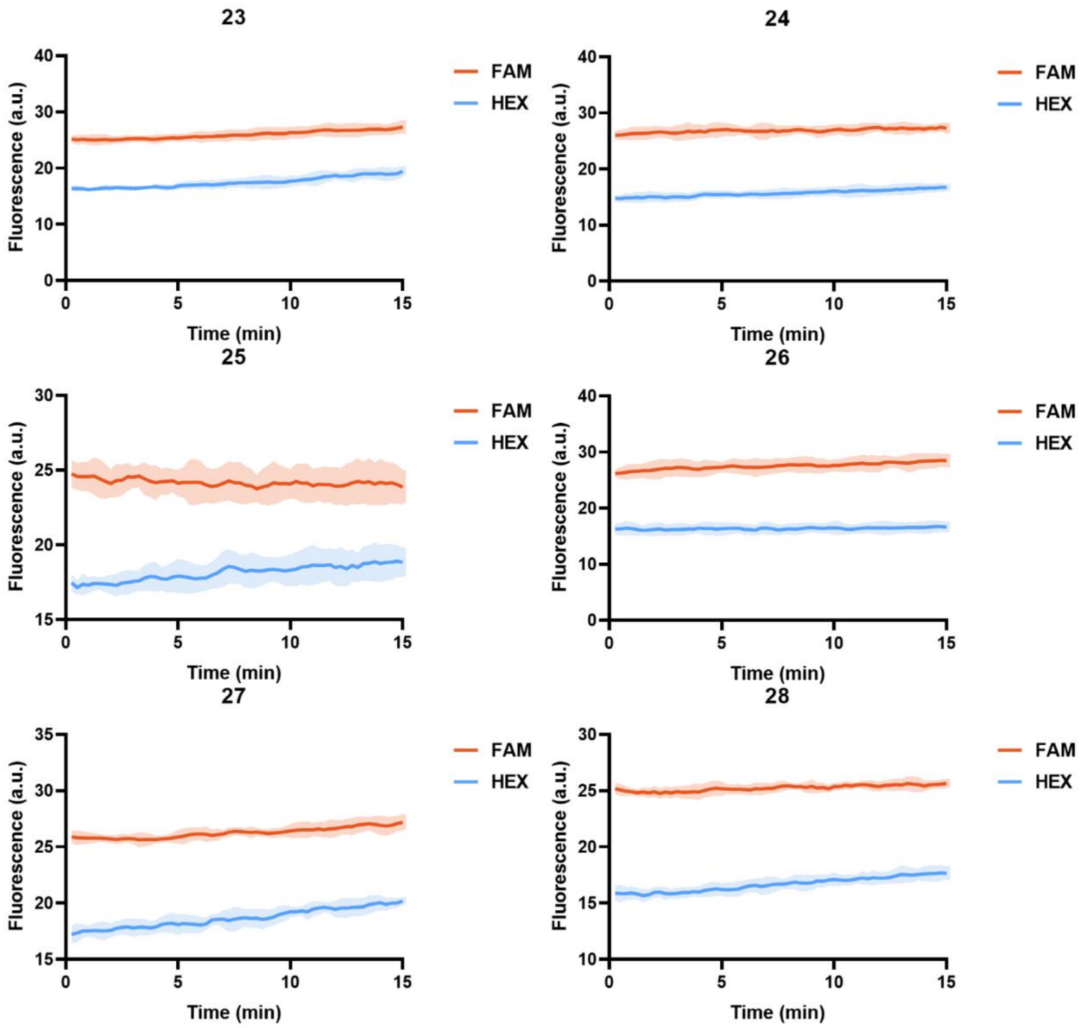

Figure S16. Fluorescence kinetics curves of cancer adjacent tissues. Condition: 10 nM PMSD and, 100 nM LCHA, 3.98 nM *Bst* polymerase. Temperature is 25 °C and buffer is Thermol pol buffer. Data are mean ± S.D. (n = 3 independent experiments).
